# Graph-based discovery and analysis of atomic-scale one-dimensional materials

**DOI:** 10.1093/nsr/nwac028

**Published:** 2022-02-26

**Authors:** Shunning Li, Zhefeng Chen, Zhi Wang, Mouyi Weng, Jianyuan Li, Mingzheng Zhang, Jing Lu, Kang Xu, Feng Pan

**Affiliations:** School of Advanced Materials, Peking University, Shenzhen Graduate School, Shenzhen 518055, China; School of Advanced Materials, Peking University, Shenzhen Graduate School, Shenzhen 518055, China; School of Advanced Materials, Peking University, Shenzhen Graduate School, Shenzhen 518055, China; School of Advanced Materials, Peking University, Shenzhen Graduate School, Shenzhen 518055, China; School of Advanced Materials, Peking University, Shenzhen Graduate School, Shenzhen 518055, China; School of Advanced Materials, Peking University, Shenzhen Graduate School, Shenzhen 518055, China; State Key Laboratory of Mesoscopic Physics and Department of Physics, Peking University, Beijing 100871, China; Electrochemistry Branch, Sensor and Electron Devices Directorate, Power and Energy Division, US Army Research Laboratory, Adelphi, MD 20783, USA; School of Advanced Materials, Peking University, Shenzhen Graduate School, Shenzhen 518055, China

**Keywords:** low-dimensional materials, 1D atomic chains, graph theory, topological classification, density functional theory

## Abstract

Recent decades have witnessed an exponential growth in the discovery of low-dimensional materials (LDMs), benefiting from our unprecedented capabilities in characterizing their structure and chemistry with the aid of advanced computational techniques. Recently, the success of two-dimensional compounds has encouraged extensive research into one-dimensional (1D) atomic chains. Here, we present a methodology for topological classification of structural blocks in bulk crystals based on graph theory, leading to the identification of exfoliable 1D atomic chains and their categorization into a variety of chemical families. A subtle interplay is revealed between the prototypical 1D structural motifs and their chemical space. Leveraging the structure graphs, we elucidate the self-passivation mechanism of 1D compounds imparted by lone electron pairs, and reveal the dependence of the electronic band gap on the cationic percolation network formed by connections between structure units. This graph-theory-based formalism could serve as a source of stimuli for the future design of LDMs.

## Teaser

Application of graph theory to inorganic compounds enables discovery of atomic-scale one-dimensional materials and helps to decipher the control of geometric features on stability and electronic structure of low-dimensional materials.

## INTRODUCTION

In 1735, driven by the belief that there should be a standard method of categorization for everything in the natural world, Carolus Linnaeus published *Systema Naturae* [1], which was the very first effort to establish scientific classifications for all species, from minerals and plants to animals, in order to understand their underlying connections. Constrained by his time, this taxonomy was mainly based on superficial observations of visible properties, shapes and traits of the objects, without consideration for the structural and genetic connectivity between these diversified species. Thus, despite its commanding and sustained success in biological kingdoms, the categorization codes are far from being applicable to the field of minerals, because our knowledge about materials and chemistries has been completely reshaped in the last century by the advent of quantum theory and the related explanation of how atoms and molecules are bonded. To date, there have been a total of over 100 000 inorganic compounds registered in the Inorganic Crystal Structure Database (ICSD) with detailed information of lattice parameters and atomic coordinates. The current categorization of these compounds is mainly based on space groups, and is incapable of evaluating the similarity and relationship among different structures. With the development of mathematical tools, there emerges an effort to encode crystal structures via graph theory [2,3], where the constructed structure graphs contain only the topological information of how atoms are connected to each other, while disregarding information about symmetry, atomic distances and bond angles that have a high reliance on the measurement of atomic position. Several pioneering works have shown the advance of structure graphs towards defining the topological features of periodic structures and predicting properties such as formation energy and band gap [4–8]. In this work, we extend those efforts to an unprecedented magnitude by demonstrating that a graph-based classification of inorganic compounds can enable us to discover potential atomic-scale low-dimensional materials (LDMs) and to capture their structure–structure and structure–property relationships.

As compared with conventional nanomaterials (nanosheets, nanofibers and quantum dots) that may possess unsaturated dangling bonds, the atomic-scale LDMs are characterized by terminated surfaces, which are chemically inert and can thus confine the intrinsic properties and functionalities of the LDMs [9,10]. In recent years, with the aid of state-of-the-art high-throughput first principles calculations, a multitude of atomic-scale LDM candidates have been discovered, predominantly in the form of two-dimensional (2D) atomic layers [11–21]. The newly established 2D materials databases [15–17] have ignited research interest in virtual screening for their potential applications as catalysts [22–24], ferromagnets [25], field-effect transistors [26] and other electronic devices [27–29], which dramatically expands the possibilities of nanoscale design in a variety of research frontiers. In contrast, it was not until recent years that other LDMs, i.e. one-dimensional (1D) atomic chains [30–37] and zero-dimensional (0D) atomic clusters [38–40], received renewed attention and enthusiasm from the academic community. Research efforts have been made with regard to the approach to top-down exfoliation of 3D bulk compounds into materials with lower dimensionality [13–17,41–43], leading to the identification of some 1D/0D compounds. However, nearly all these works only focused on the dimensionality determination schemes with an aim to discover the low-dimensional compounds, while systematic investigation is absent concerning what kind of structural motifs they tend to possess and how their physical/chemical properties are affected by geometry. It is worth emphasizing that symmetry is often broken in LDMs due to the abundance of edge sites and the nano-size effects. Therefore, traditional symmetry-based classification methods could only provide very limited information for the understanding of their structures and the prediction of properties. The lack of a standardized protocol to classify and analyze geometric structures has proven to be a technical barrier to exploring their chemical space and intrinsic characteristics. Moreover, while 1D Si/Ge/III-V nanowires [44–46] are widely regarded as one of the most competitive channel materials of the next-generation transistors to supplant the Fin channels, dangling-bond-free semiconducting 1D atomic chains, if available, might be expected to outperform these nanowires in a gate-all-around device. In this context, it would be of critical importance to establish a correlation between intrinsic properties and salient topological features of the atomic-scale 1D materials.

Herein, a novel protocol based on graph theory and density functional theory (DFT) calculations is conducted with the aim of structural classification and topological analysis for atomic-scale LDMs. 1D materials are of special interest because their distinct difference from their 2D counterparts may have broad appeal to researchers in the area of electronic devices. By categorizing the structural blocks in bulk compounds retrieved from ICSD, 244 candidate 1D compounds are discovered and nearly half of them are predicted to be potentially exfoliable from parent bulk crystals. The topological classification approach has enabled us to categorize 1D compounds into chemical families with distinct bonding character and atomic size difference. We present specific cases to illustrate the structural inheritance of 1D structures from 2D compounds, and highlight the role of the lone electron pair (LEP) on self-passivation at edge sites. Graph theory is further employed to group 2D and 1D compounds with similar cation-connection motifs, from which we identify a statistical correlation between the atomic networks and their electronic band structures. The findings in this work shed light on a new paradigm wherein the structure-property relationships in materials are captured by insights into structure units and their specific connection patterns.

## RESULTS AND DISCUSSION

### Workflow

**Figure 1.**
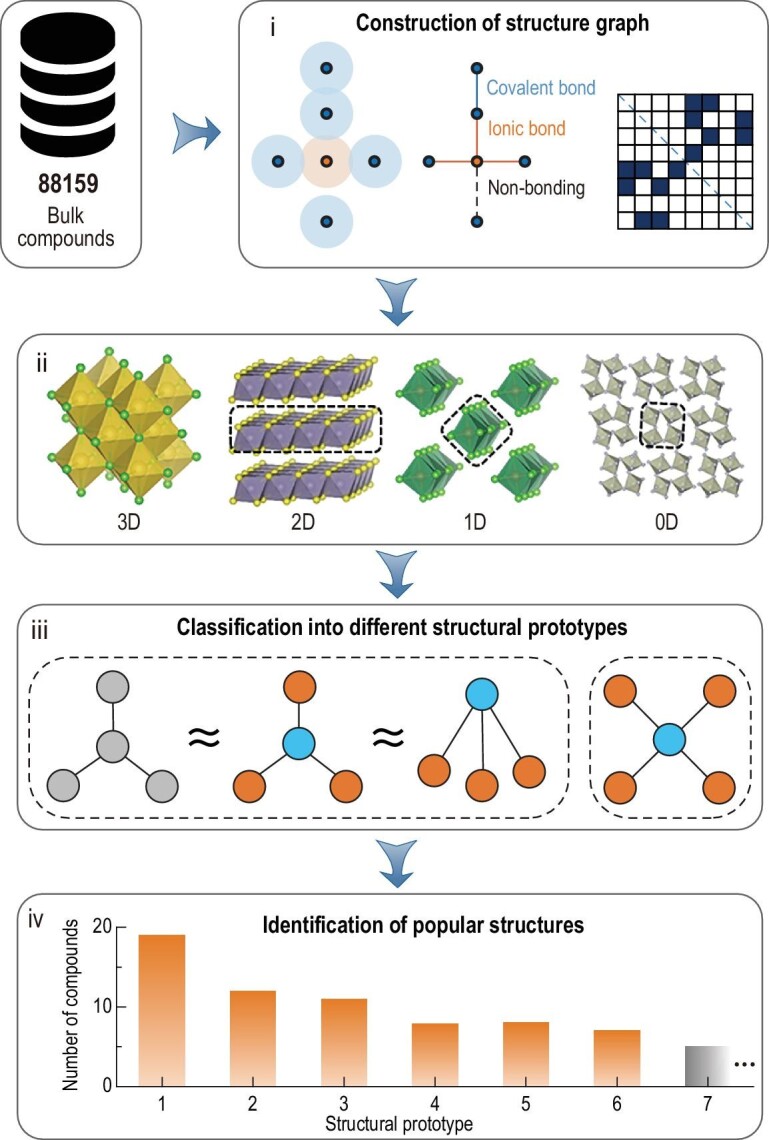
An overview of the workflow for LDM identification and classification. Four main steps are included: (i) construction of structure graphs for bulk compounds; (ii) identification of the chemically connected structural blocks with different dimensionalities; (iii) classification of the isolated structural blocks according to their graphs; (iv) statistics on the structural prototypes of 1D compounds, e.g. the number of compounds for each kind of structure.

In structure graphs, atoms are represented as nodes and the direct chemical interactions are abstracted as connections between these nodes. The graph-based classification of materials into different geometric categories is a rigorous and unbiased approach to investigating structural prototypes, as long as the atomic bonding is properly defined. To discover atomic-scale LDMs from bulk compounds, we first acquire the structure graphs of the available compounds in ICSD (Fig. 1). By discarding error items as well as materials with fractional occupancy at lattice sites, there remain a total of 88 159 unique compounds. For each compound, neighboring atoms are searched using the covalent, ionic or metallic radii (Table S1) of the corresponding atoms. The choice of which kind of radius to adopt is in accord with their ionization energies. The atomic adjacency matrix is then constructed, which presents a portrait of the compound and serves as the representative of structure graph for the identification of exfoliable materials. We use this adjacency matrix to determine the dimensionality of the chemically connected blocks in the bulk material. The 2D, 1D and 0D structural blocks are successfully identified and isolated to produce a library of LDMs, after which we apply graph theoretical analysis again to group them into different structural prototypes. *Ab initio* molecular dynamics simulations are employed to assess the dynamic stability of the 1D compounds and filter out those showing a high propensity to decompose at room temperature. The above procedures yield a total of 244 stable unique 1D compounds, which are categorized into 138 structural prototypes.

Notably, a subset of the identified 1D compounds has been previously predicted computationally [14,42,43], despite the difference in both prediction algorithm and the criterion for atomic connectivity. In this work, the chemical bonding is determined by a prior classification of covalent, ionic and metallic interactions between possible neighboring atoms, which is consistent with the fact that bond lengths can vary according to their covalency, ionicity and metallicity. The proper choice of atomic radii in our identification protocol can help assess atomic interactions in a relatively objective way, and will therefore offer ample opportunities to discover potential LDMs. Nevertheless, we believe that only when a sufficiently large set of LDMs are synthesized experimentally can we compare the reliability of different identification methods. In this sense, detailed comparison between this graph-theory-based scheme and former LDM identification protocols is beyond the scope of this work.

### Data set of 1D materials

**Figure 2.**
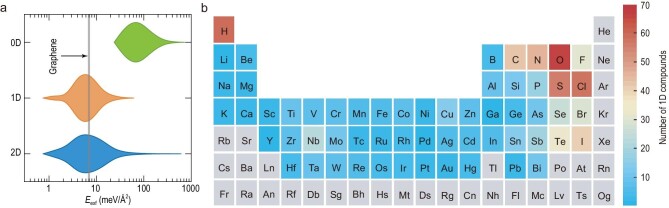
Exfoliation energy of LDMs and distribution of compositions in 1D compounds. (a) Violin plots of the exfoliation energy per surface area for 2D, 1D and 0D compounds. The gray line indicates a threshold defined by the value of *E*_exf_ for graphene. (b) Number of 1D atomic chains containing a given element. Elements in gray are not present in any compound.

The exfoliation energy *E*_exf_, defined as the energy difference between the LDMs and their parent bulk compounds [47,48], is computed to evaluate the feasibility of exfoliation. Figure 2a shows the violin plot of the calculated *E*_exf_ for 2D, 1D and 0D compounds, which indicates the kernel density of distribution. A considerable number of 2D and 1D materials require relatively little energy to be exfoliated, whereas the exfoliation of the 0D atomic clusters tends to incur a large energy penalty. The distributions of exfoliation energy for 2D and 1D compounds are quite similar. Remarkably, we have uncovered 105 1D materials that are likely as exfoliable as typical 2D materials (the threshold of *E*_exf_ is chosen from graphene). We are therefore confident to carry out the systematic analysis of the largely unexplored realm of 1D atomic chains. Figure 2b summarizes the number of 1D compounds containing a specific chemical element. We note that the composition of 1D compounds can span across nearly the whole periodic table, reflecting a high chemical diversity. Typical non-metal elements, such as O, S and Cl, have shown their copious presence in 1D structures, while elements for metal cations are fairly diversified, with Nb being slightly more common than other transition metal cations.

**Figure 3.**
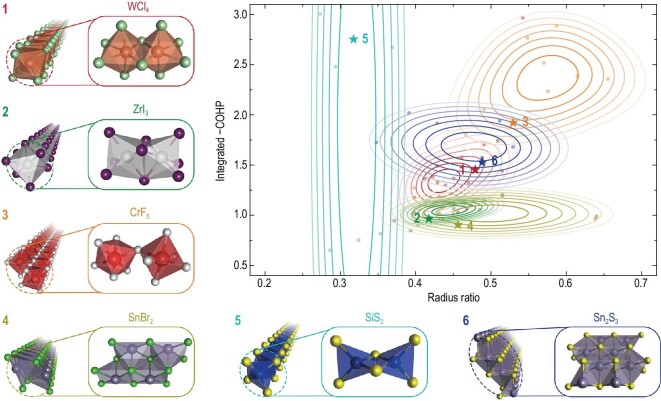
The six most popular structural prototypes of 1D atomic chains. The different chemical spaces of these prototypes are reflected by the kernel density distributions of integrated −COHP (indicative of bonding interaction) and radius ratio (*r*_cation_/*r*_anion_, indicative of atomic size effect) for the cation-centered coordination polyhedra.

The structures of the six most popular structural prototypes, as identified by the graph-based classification, are displayed in Fig. 3. In Table S2, we present examples of other representative structural prototypes. To describe the 1D structure chemistry, we use two basic descriptors corresponding to the bonding interaction and the atomic size effect, respectively. The former is represented by the integrated crystal orbital Hamilton population (COHP) according to the DFT-computed electron density (see Methods), while the latter is quantified by the radius ratio between cations and anions in the cation-centered coordination polyhedra. As is evident from Fig. 3, the distributions of the six structural prototypes in the chemical space are quite different from each other. For example, compounds featured by face-sharing MX_6_ (M: cation; X: anion) octahedra (Group 2) are mostly iodides and bromides, which consistently have weak bonding interactions and relatively small radius ratio (*r*_cation_/*r*_anion_). This kind of structural motif coincides with the tendency of I^−^ and Br^−^ ions to be compactly packed due to their large radii and weak electrostatic repulsion. In contrast, the vertex-sharing prototypes (Group 3) are in the top-right of the diagram, which is associated with the predominance of F^−^ and O^2−^ anions in these compounds. The high oxidizing ability of F and O gives rise to strong charge localization, which, along with the short metal-F/O bond lengths, imposes significant electrostatic interactions between the cations, thus rendering the vertex-sharing architecture more favorable than close-packed ordering. As intermediates between Group 2 and Group 3 compounds, most of the edge-sharing Group 1 compounds contain Cl^−^ anions and cover a region in the middle of the panel. Hence, we can expect there to be a subtle but definite correlation between the connection patterns (vertex/edge/face-sharing) of the structure units [49] and the chemical space of the corresponding 1D compounds. Moreover, we observe that compounds with tetrahedral coordination polyhedra (Group 5) distribute in a wide range of bonding interactions, but with distinctively small radius ratio. This result essentially follows Pauling's first rule, which establishes the dominant role of radius ratio in regulating the coordination number of cations. Altogether, the classification result has revealed the ability of structure graphs to capture and differentiate the major features of 1D compounds, thus constituting a new informatics tool to facilitate the analysis of LDMs.

Despite the diverse nature of cation-connection motifs, most of the popular structural prototypes (Table S2) comprise regular coordination polyhedra (octahedral or tetrahedral). The absence of dangling bonds on the cations and the close-shell electronic structure of the anions can endow their parent bulk materials with a highly anisotropic character, whereby physical and chemical properties can remain unaffected by grain boundaries and surfaces parallel to the 1D chain. As long as they have suitable band gaps, it would be highly desirable to fabricate electronic devices with these compounds, which can significantly suppress the scattering from surface roughness and trap states [50,51]. For compounds with irregular coordination polyhedra, one would expect that the unsaturated dangling bonds would impair their stability. However, this is not entirely true if we refer to the calculated exfoliation energy. Illustrative examples are Group 4 and Group 6 compounds, for which the exfoliation energies fall into the 7−19 meV/Å^2^ range (Table S3), larger than other structural prototypes but not to a significant extent. Our results demonstrate that the data mining of topological information can stimulate the discovery of materials with unusual structures, which may complement our understanding of LDMs.

Unlike 1D atomic chains, isostructural 0D compounds are relatively rare; in other words, versatile structures can be obtained with similar constituent elements (Table S4). This is attributable to the higher flexibility in the number of constituent atoms in a cluster, as imposed by the removal of periodic boundary conditions in all directions. The repository of 0D materials shows a high prevalence for covalent bonding between main-group elements, including boron, carbon and phosphorus (Tables S5–S7), with numerous architectures existing in various compositions. The assembly of these atomic clusters into their parent bulk materials can present fascinating examples for studying the packing of fullerene-like molecules [38,39].

### Self-passivation in atomic chains

**Figure 4.**
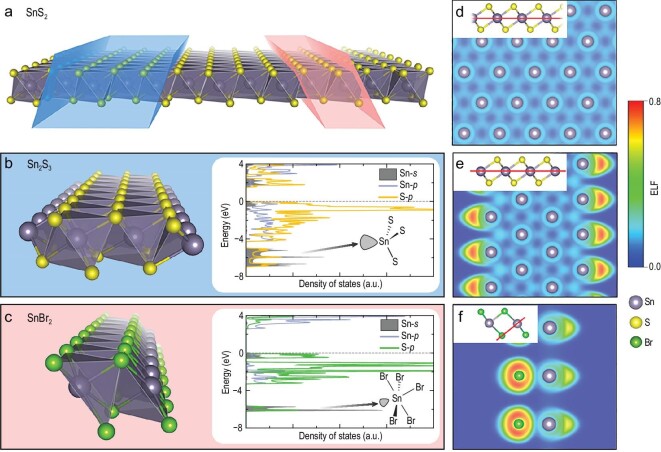
Self-passivation via LEP on Sn^2+^. (a) Structure of SnS_2_ monolayer. The blue and red frames denote the structural inheritance of Sn_2_S_3_ and SnBr_2_ from SnS_2_, respectively. Structures of (b) Sn_2_S_3_ and (c) SnBr_2_ atomic chains and their electronic DOS. Fermi level is set to zero. States in the energy range of −7 ∼ −5 eV correspond to the 5*s* LEP on Sn^2+^ ions. ELFs of (d) SnS_2_, (e) Sn_2_S_3_ and (f) SnBr_2_ in the planes indicated by the red lines in the insets. Local maximum of ELF around the Sn atom defines the location of the LEP.

The semi-regular coordination polyhedra in some 1D atomic chains have led us to explore the self-passivation mechanism of their unsaturated dangling bonds, which could create the possibility of manipulating the dimensionality of materials. To this end, we screen the collection of 2D and 1D compounds for cases where the 1D structure is identical to a subgraph of any of the 2D structures. This criterion is met by 1D Sn_2_S_3_ and SnBr_2_ atomic chains (Fig. 4a–c), both of which are well correlated with the 2D SnS_2_ structure. Other 2D–1D correlated structures are shown in Fig. S9. As compared to the SnS_6_ octahedron in SnS_2_, the edge of Sn_2_S_3_ features trigonally coordinated Sn^2+^ (valency is deduced from Bader charge in Fig. S10), while in SnBr_2_, Sn^2+^ ions sit at pyramidal sites. Apparently, each of the atomic chains adopts a structure that can be regarded as truncated from the 1T-phase SnS_2_, which leaves unsaturated dangling bonds free to interact with external atoms. To clarify the electronic origin of the feasibility for isolating Sn_2_S_3_ and SnBr_2_ atomic chains from bulk phases, we calculated the density of states (DOS) localized on different atomic sites, as shown in the insets of Fig. 4b and c. The Sn 5*s* orbitals occupy deep energies far from the valence band maximum (∼6 eV apart), indicating that the LEP of the Sn^2+^ ion is relatively non-labile. To trace the location of the LEP, we calculated the electron localization function (ELF) [52] for Sn_2_S_3_ and SnBr_2_ (Fig. 4d–f), with SnS_2_ shown for comparison. A local maximum of the ELF is observed at the edges of the atomic chains, pinpointing the lobe-like LEP that deviates from the central atom nucleus of Sn^2+^. We note that the LEP is stereochemically active and therefore participates in the coordination polyhedron of Sn^2+^, resembling the cases of Pb^2+^ and Bi^3+^ in lone pair containing compounds [53].

Due to the spherically symmetric nature of *s* orbitals, there must be hybridization between Sn 5*s* and other states with *p* character in Sn_2_S_3_ and SnBr_2_, otherwise the LEP will not form a lobe shape [54]. As can be inferred from the significant overlap between Sn-*s* and S/Br-*p* orbitals in the DOS (Fig. 4b and c), the Sn 5*s* and anion *p* states are energetically degenerate in these atomic chains. The COHP plot shown in Fig. S11 reveals a bonding-antibonding fashion between Sn and S/Br in the energy range of −7 ∼ −5 eV (bonding) and just below the Fermi level (antibonding), suggesting that the majority of Sn 5*s* states are involved in bond formation with S/Br-*p* states. The charge density (Fig. S12) between −7 and −5 eV also shows a considerable proportion of electrons distributed between Sn and S/Br. These results demonstrate that in addition to fulfilling the role of a ligand for the undercoordinated Sn^2+^ ion, the LEP in Sn_2_S_3_ and SnBr_2_ can endow high stability to the edges of both materials via strong coupling with the anion *p* states. It is therefore expected that edge doping of main-group elements with an LEP, such as Sn^2+^, Pb^2+^ and As^3+^, will stabilize the 2D nanoribbons and can therefore serve as a powerful strategy for harnessing the dimensionality and chemical properties of LDMs.

### Correlation between cation connectivity and electronic band gap

**Figure 5.**
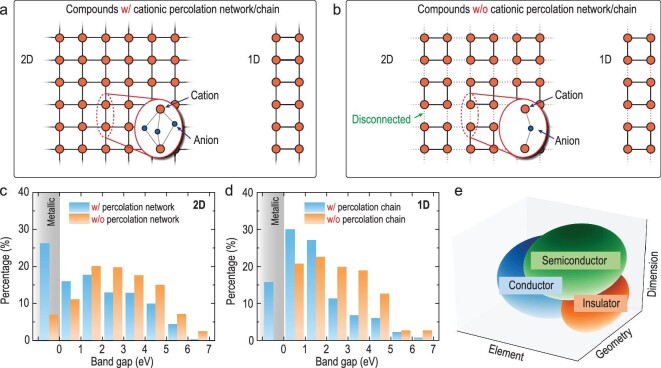
Influence of cation connectivity on electronic band gap. Schematic illustration of 2D and 1D structures (a) with (w/) or (b) without (w/o) a cationic percolation network/chain. The distribution of DFT-predicted band gaps for (c) 2D and (d) 1D compounds, each divided into two groups by the existence of a cationic percolation network. (e) Modulation of electronic structure by tuning the constituent element, geometry and dimensionality of the material.

Graph theory can also be introduced to unravel the structure–property relationships of LDMs. Here, we examine how dimensionality and cation connectivity exert an influence on the electronic band structure of 2D and 1D materials. We note that the cation-centered coordination polyhedra embrace a much larger proportion of vertex-sharing linkages in LDMs (Fig. S13) than in bulk compounds that cannot be partitioned into LDMs. Heuristically, an intimate connection between cations can be defined as either a direct connection via metallic bonds or face/edge-sharing linkages of the corresponding polyhedra. In contrast, vertex-sharing linkage underlies the disconnection between adjacent cations. We discover many 2D (Fig. S14) and 1D (e.g. WCl_4_, ZrI_3_ and SnBr_2_) materials in which the interconnected structure units form a percolation network (for 1D materials it will be a percolation chain). This percolation property can serve as a powerful classification rule to distinguish between densely and loosely packed sublattices of cations (Fig. 5a and b). Notably, over half of the 2D and 1D compounds are found to possess a cationic percolation network (Fig. S16). Although the ratio of vertex-sharing linkage is nearly the same at both dimensionalities, a higher fraction of 2D materials, compared to 1D atomic chains, are found to possess such a network. This result implies that cation connectivity can be biased by the dimensionality of compounds. We would like to stress that the above classification scheme is not guided by measured interatomic distances between the cations but by structure graphs of the LDMs, because the distances are element specific and coordination number dependent, which precludes the use of a global threshold for the identification of connections.

Figure 5c and d present the distribution of band gaps for 2D and 1D compounds with (w/) and without (w/o) a cationic percolation network. As apparent from the histograms, the group featuring a cationic percolation network tends to yield a smaller band gap than its counterpart. The existence of a cationic percolation network corresponds to the higher packing density of the cation-centered coordination polyhedra and therefore leads to a closer proximity between these structure units than the configuration without a percolation network. In this scenario, we can expect there to be a collection of interconnected atoms with a high degree of overlap in the atomic orbitals, whereby the bandwidth would be widened and the band gap narrowed. Compounds without a band gap are much scarcer in 1D atomic chains than in 2D counterparts, which can be associated with the higher susceptibility of 1D compounds to structural distortion that leads to splitting of degenerate states. It is worth mentioning that there exist compounds where some of the cations adopt vertex-sharing linkage, while other cations share multiple anions. As long as the cation-centered polyhedra or cations themselves form penetrating paths throughout the compound, we should regard them as having a cationic percolation network, even though not all cations are involved in its formation. This is based on the assumption that the dense packing of the constituent cation-centered polyhedra in the percolation network would lead to a high degree of electron-cloud overlap and consequently affect the electronic states near the Fermi level, no matter whether there were cations outside this network or not.

Although many studies on atomic-scale LDMs have shown the dependence of their electronic properties on the constituent elements and structure units [22–25,27,28], none show the essential role played by dimensionality and cation connectivity. The above results demonstrate that a combination of elemental, geometrical and dimensional modulation can be incorporated to mediate the electronic structure of LDMs (Fig. 5e), and that the exploration of cationic percolation networks represents a viable avenue for the design of low-dimensional conductors, semiconductors and insulators for use in the fabrication of electronic devices.

## CONCLUSION

Through the application of graph theory, which permits the encoding of topological information for all structures, this work presents a major step towards the comprehensive and systematic discovery and analysis of atomic-scale 1D compounds. The algorithmic approach is applied to the experimentally known compounds from ICSD, yielding a variety of structural blocks with different dimensionalities. Many of them are expected to be exfoliable from bulk phases, and their isolation into LDMs could introduce a promising platform to expedite experimental investigations into quantum confinement effects. The full collection of 1D atomic chains displays a fascinating structural diversity, with various prototypes spanning a broad chemical space. We find that the types (octahedron/tetrahedron) and connection patterns (vertex/edge/face-sharing) of the structure units in 1D compounds have a non-trivial correlation with the chemical space, where the bonding character and atomic size difference represent two major contributing factors. Enlightened by examples of 1D compounds whose structures can be truncated from certain prototypes of 2D compounds, we propose a novel strategy, based on LEPs of *s* orbitals, to self-passivate the dangling bonds, thus offering opportunities for the control of dimensionality. Given the low-lying energy levels of these LEPs, our strategy can overcome the shortcomings of conventional nanoribbons and nanorods, such as poor stability and the formation of unfavorable edge states. We also employ structure graphs to distinguish between 2D/1D compounds with and without a cationic percolation network. It is revealed that the emergence of such a network would generally be accompanied by a relatively narrow electronic band gap, implicating the essential role of linkages between cation-centered coordination polyhedra in deciding electronic structure. Together, these insights suggest that the physical properties of atomic-scale 1D materials are to a large extent dependent on their topological features, which, as portrayed in the structure graphs, provide a new perspective on the characterization of structures with reduced dimensionality.

## METHODS

### DFT calculations

Structural optimization and energy calculations were performed via PWmat code [55,56], which is a plane wave pseudopotential package based on DFT and accelerated by graphics processing unit (GPU) architecture. The Perdew–Burke–Ernzerhof (PBE) [57] generalized gradient approximation (GGA) functional and NCPP-SG15-PBE pseudopotential [58,59] were used. The electron wave functions were expanded by plane waves with cut-off energies of 50 Ryd. The convergence tolerance for total energy and residual force on each atom during structure relaxation was set to 10^−5^ eV and 0.02 eV/Å, respectively. In all the calculations except for atomic clusters, the Brillouin zone was sampled by Monkhorst-Pack *k*-point grid [60] with a total of at least 1000/(the number of atoms per cell) points for all directions. To complement the deficiency of DFT in treating dispersion interactions, a van der Waals correction term developed by Grimme (D3) [61] was adopted. Spin polarization was taken into consideration and the ferromagnetic configuration was set as the initial magnetic structure. To avoid spurious interactions between periodic images, a space vacuum of at least 15 Å was placed between the periodic images of atomic layers, chains and clusters. Dipole correction was applied for all the polar LDMs [62].

The calculations of Bader charge, electron density distribution and electron localization function were performed on the Vienna *ab initio* simulation package (VASP) [63,64] using the plane-wave projector-augmented wave method [65] and GGA-PBE functional. Energy cut-off was set to 520 eV and the *k*-point sampling was the same as that in PWmat calculations. The structures of representative 1D compounds were optimized by VASP before the electron density calculations. Based on the calculated DOS, the COHP [66,67] was obtained using Local Orbital Basis Suite Towards Electronic-Structure Reconstruction (LOBSTER) code [68,69]. COHP can quantitatively interpret bonding and antibonding contributions according to the generated overlap population-weighted DOS between atoms. The bonding interaction for each pair of neighboring atoms can be indicated by the integration of –COHP from the lowest energy level of the valence electrons to the Fermi level of the compound [70].

## Supplementary Material

nwac028_Supplemental_File
