# Supplementary material for: Graph-based discovery and analysis of atomic-scale one-dimensional materials
Source: Natl Sci Rev. 2022 Feb 26;9(6):nwac028. doi: 10.1093/nsr/nwac028 (PMC9170357; doi:10.1093/nsr/nwac028)
Supplement: nwac028_Supplemental_File [file nwac028_supplemental_file.pdf]

Supplementary data for

Graph-based discovery and analysis of atomic-scale one-dimensional materials

Shunning Li,[1]† Zhefeng Chen,[1]† Zhi Wang,[1]† Mouyi Weng,[1]† Jianyuan Li,[1] Mingzheng Zhang,[1] Jing Lu,[2] Kang Xu,[3] Feng Pan[1]*

[1]School of Advanced Materials, Peking University, Shenzhen Graduate School, Shenzhen 518055, China.
[2]State Key Laboratory of Mesoscopic Physics and Department of Physics, Peking University, Beijing 100871, China.
[3]Electrochemistry Branch, Sensor and Electron Devices Directorate, Power and Energy Division, US Army Research Laboratory, Adelphi, MD, USA.

*Correspondence: panfeng@pkusz.edu.cn
†These authors contributed equally to this work.

Database of existing bulk compounds

The database of bulk compounds used in this study is obtained from the Inorganic Crystal Structure Database (ICSD, version 2019), with a total of 186,039 data entries. Among them, 89,135 compounds with fractional occupancy at lattice sites are excluded from the calculations. We find that some of the data entries contain contradictory information in chemical composition, while some others have missing atoms in the atomic-coordinate data block. These entries are considered as error items and therefore filtered out from the database of bulk compounds (Fig. S1). Moreover, there are several compounds whose crystal structures experience dramatic changes after structural optimization using density functional theory (DFT) calculations. To prevent the inconsistency between the structures of low-dimensional materials (LDMs) and their parent bulk crystals, the graph-theory-based algorithm for structural block identification is carried out after the DFT structural optimization of the corresponding bulk compounds.

Criterion for atomic connectivity

The chemical bonding character of each pair of neighboring atoms is determined by their ionization energies. If both atoms exhibit ionization energies of at least 8.1517 eV (the value of Si), we use covalent radii to assess their connectivity. When both ionization energies are lower than 8.1517 eV, metallic radii are employed. The rest of the cases correspond to ionic bonding, in which ionic radii are adopted. Note that any nonmetal atom could only be negatively charged when forming ionic bond with a metal atom, and that the positively charged nonmetal atoms will appear in the covalent bonding cases according to the above protocol. Table S1 tabulates the covalent, metallic and ionic radii used in this work, which are extracted from literature [1-3]. It should be pointed out that although the ionic radii can vary with the valence states, here we only employ the maximum value for the evaluation of atomic connectivity. Since the ionic radii of H, C, Si, P and As are unavailable in the literature, we carry out systematic searches in ICSD for ionic compounds containing these elements, and establish empirical ionic radii for them on the basis of interatomic distances and the known radii of other elements. Figure S2 shows the comparison between the predicted interatomic distances using the deduced atomic radii of the above elements and the corresponding experimental data. The chemical bond of an atom pair is heuristically identified when the interatomic distance is smaller than the sum of radii multiplied by 1.15, which is essential for including the effect of bond-length fluctuations while not introducing too much empirical input.

The value of the multiplier (1.15) for bond length cutoff was chosen under the following consideration. As shown in Fig. S3a, the interatomic distance divided by the sum of the corresponding atomic radii, here referred to as normalized distance, will theoretically reach a peak at ~1.0. This corresponds to the contribution from the first nearest neighbors (1st NN) of a central atom. Another peak corresponding to the 2nd NN will

appear at larger distance. Considering the distribution of normalized distance for the whole collection of bulk compounds, it is not surprising to find that the 1st NN peak will partially overlap with the 2nd NN peak. In this overlap region, the number of bonds is relatively small (valley of bond length distribution). Therefore, in the total number of bonds *vs.* normalized distance plot, we will find a gentle-slope region in between two steep regions. The value of the multiplier was chosen at this region (Fig. S3b) to guarantee that most of the 1st NN and few of the 2nd NN are considered for the construction of structure graphs. Larsen et. al. recently proposed that dimensionality classification of the structural blocks in solid-state materials can be accomplished by a scoring parameter derived from the threshold values of the multiplier for bond length cutoff [4]. Our choice of 1.15 is close to the contour line of $s(k_1, k_2) = 0.5$ in the above work. To ensure consistency in the codes, the value of multiplier is kept fixed for both dimensionality identification and topological classification of the structures.

Identification and classification of structural blocks

The topological structure of any bulk compound can be depicted by a simple undirected graph. Atoms are represented by small circles, and chemical bonds by lines that connect the corresponding circles. The structure graph of NaCl is exemplified in Fig. S4. Both Na and Cl atoms in a unit cell are taken as the starting point for constructing the subgraphs, which merge into a complete graph where all atoms in the unit cell are involved. The shortest path between any two nodes is called a geodesic and its length defines the distance between nodes. Atoms linked to the original Na/Cl have a distance of 1 to the central atoms, and are therefore described as the 1st NN. Atoms with distance of 2, 3 and so on are the 2nd NN, the 3rd NN and so on. Each structure graph is represented by an adjacency matrix defined as follows

$$\boldsymbol{A}_{jk} = \begin{cases} 0 & \text{... if } j = k \\ 1 & \text{... if } j \text{ and } k \text{ are connected} \\ 0 & \text{... if } j \text{ and } k \text{ are not connected} \end{cases} \tag{S1}$$

This matrix is used for evaluating the isomorphism among different structures. Two structures may be considered as topologically identical whenever there is a bijective mapping between their structure graphs. This mapping is performed on all the subgraphs, which can assist in the automatic deduplication of crystal structures and make possible the classification of structural prototypes. For example, the matrix representations of 1T and 2H phases of MoS_2 monolayer can be distinguished from each other. Figure S5 shows the 1st and 2nd nearest neighbors of 1T and 2H phases of MoS_2 monolayer, as well as their corresponding adjacency matrices for the subgraphs. The connection between the central and the 2nd NN are different, which can be directly reflected by the highlighted parts of their adjacency matrices. Because their subgraphs are not isomorphic, the 1T and 2H phases of MoS_2 monolayer will belong to different prototypes according to our graph-based classification. Therefore, we believe that the graph-theory-based approach in this work is capable of capturing the geometric

difference and portraying the key features of LDMs. This approach is particularly important for 1D and 0D compounds, since their symmetry can generally vary in an easier way than bulk structures. Atomic connectivity will likely exert much greater impact than symmetry on the properties of 1D atomic chains. Nevertheless, we would like to note that there are still some cases where two structures with isomorphic subgraphs are quite distinct from each other, generally due to the difference in symmetry or bond angles. For example, $NbCl_4$ and $ZrCl_4$ in Table S2 display the same subgraph but apparently not the same structure. This stems from the fact that their MX_6-interconnection edges are distributed in different patterns. Given that the chemical bonds of Nb-Cl and Zr-Cl, as well as the ionic radii of Nb and Zr, are relatively similar to each other when compared to those pairs that belong to two different groups, we can assure the capability of graph-based classification in differentiating the major features of 1D compounds.

To determine the chemically connected structural blocks in bulk materials, a structure graph based on the 5×5×5 supercell of the compound is employed. All atoms in the supercell are included as nodes. The corresponding adjacency matrix can be transformed into block diagonal matrix. For example, if matrix $\boldsymbol{A}$ can be blocked into three smaller square matrices $\boldsymbol{A_1'}$, $\boldsymbol{A_2'}$ and $\boldsymbol{A_3'}$, with zero matrices $\mathbf{0}$, then we have:

$$\boldsymbol{A'} = \boldsymbol{Q}^{-1}\boldsymbol{A}\boldsymbol{Q} = \begin{pmatrix} \boldsymbol{A_1}' & \mathbf{0} & \mathbf{0} \\ \mathbf{0} & \boldsymbol{A_2}' & \mathbf{0} \\ \mathbf{0} & \mathbf{0} & \boldsymbol{A_3}' \end{pmatrix} \qquad \text{(S2)}$$

From the reduced matrices $\boldsymbol{A_1'}$, $\boldsymbol{A_2'}$ and $\boldsymbol{A_3'}$, we can identify the structural blocks and determine their dimensionality. We consider an example where graphene is extracted from the structure of graphite, as shown in Fig. S6. Supercell is built and converted into a different structure graph from that of the unit cell. The new structure graph is represented by an adjacency matrix of order 5×5×5×N (N is the number of atoms per unit cell). After matrix transformations, a block diagonal matrix is obtained, in which each block corresponds to an assembly of chemically connected atoms in the supercell. The structural blocks can be uniquely constructed according to the matrix and are isolated into low-dimensional structures. It is worth noting that there exist cases where the structural blocks are off-stoichiometric with respect to the parent bulk compounds. The derived LDMs tend to undergo significant structural reconstruction due to the change in valency. Therefore, in order to avoid unrealistic predictions, these cases are left out of consideration in the present work.

The dimensionalities of LDMs are distinguished via the following procedure:

(1) Select a random atom A_0 in the middle of the 5×5×5 supercell and search all its periodically equivalent atoms $\{A_i\}$ ($i \geq 1$) inside the structural block where A_0 resides.
(2) If there is no periodically equivalent atom for A_0, the structural block is zero-dimensional.

(3) Let (x_i, y_i, z_i) be the coordinate of atom A_i. If for all atoms in $\{A_i\}$ ($i \geq 0$), the coordinates of every three atoms (i, j and k) satisfy $\boldsymbol{d}_{ij} = m\boldsymbol{d}_{ik}$, where $\boldsymbol{d}_{ij} = (x_j - x_i, y_j - y_i, z_j - z_i)$ and m is an integer, the structural block is one-dimensional.

(4) If (3) is not satisfied, the plane defined by these three atoms (i, j and k) will correspond to the following equation:

$$\begin{vmatrix} x - x_i & y - y_i & z - z_i \\ x_i - x_j & y_i - y_j & z_i - z_j \\ x_i - x_k & y_i - y_k & z_i - z_k \end{vmatrix} = 0 \tag{S3}$$

If for all other atoms in $\{A_i\}$ ($i \geq 1$), their coordinates satisfy the above equation, the structural block is two-dimensional.

(5) If (4) is not satisfied, the structural block is three-dimensional.

Sn_4P_3 and La_2PBr_2 (Fig. S7) are two representative compounds that according to our calculations, exhibit the potential for exfoliation into 2D atomic layers, while not yet in the list of 2D compounds in previous studies. Conventional approaches rely on the van der Waals radii, which tend to classify the Sn-Sn and Br-Br bonds as connected [5], contradicting the DFT calculated electrostatic potentials (insets in Fig. S7) which reveal a relatively weak interaction between neighboring structural blocks in both materials and suggest the possibility of exfoliation. We believe that a prior classification of covalent, ionic and metallic interactions between possible neighboring atoms can help determine the proper choice of atomic radii, thus yielding the bonding cutoff distances in a precise way. All the representative 1D materials (in 10 most popular structural prototypes) are tabulated in Table S2.

Calculation of exfoliation energy

The exfoliation energy per surface area for the LDMs is calculated as follow:

$$E_{\text{exf}} = \frac{E_{\text{iso}} - E_{\text{bulk}}/n}{S_{\text{suf}}} \tag{S4}$$

where E_{iso} is the total energy of the LDM per unit cell, E_{bulk} is the total energy of the parent bulk compound consisting of n structural blocks (each structural block corresponds to a unit cell of the LDM), and S_{suf} is the effective surface area of the LDM. For 2D materials, the S_{suf} is defined as double the in-plane area (S) of the bulk unit cell. To calculate the effective surface area of 1D and 0D compounds, we treat an atomic chain as a cylinder and an atomic cluster as a sphere. Their volumes are estimated by dividing the volumes of their parent bulk compounds by n. The surface area is then derived according to the calculated volume, as shown in Fig. S8. In Table S3, we tabulate the calculated exfoliation energy of all the representative 1D materials listed in Table S2.

Identification of structural correlation between 2D and 1D structures

The identification of cases where a 1D structure is identical to a subgraph of any of the 2D structures can be accomplished by the following procedure. First we select 2D structural prototypes consisting of less than 20 atoms in a unit cell, and construct a 4 × 4 supercell for each structure. All possible subgraphs of interconnected atoms in a 2D structure are then enumerated and compared with those of 1D structures. When the structure graph of the 2D structure is found isomorphic to any of the 1D structural prototypes, we shall say that this 1D structure can probably be truncated from a 2D material. For example, when investigating the correlation between the structures of 1T-phase SnS_2 atomic layer and the edge-sharing prototype (Group 1) of 1D atomic chains, we will first construct the 4 × 4 supercell for SnS_2 and select one of the atoms (e.g. Sn atom) as the starting atom. Then one of its neighboring atoms (S atom) is randomly chosen as the second atom, and the neighboring atom of the first or second atom is picked out as the third one, and so on. In this atomic sequence, if there exist two atoms that are neighbors across the boundary of the supercell, the corresponding structure graph of this atomic sequence will be constructed and compared with that of WCl_4 (1D material of Group 1). Similar process is done for all the six most popular 1D structural prototypes. In this way, we found that 2D SnS_2 can be truncated into 1D structures of Group 1, 4 and 6. It is worth noting that the truncation of the cation-centered coordination polyhedra in 2D compounds will result in a lower coordination number of the cations in the corresponding 1D structure. The related 2D and 1D structures are displayed in Fig. S9.

Calculation of electronic structure

Bader charges of Sn, S and Br ions in 2D-SnS_2, 1D-Sn_2S_3 and 1D-$SnBr_2$ are provided in Fig. S10. The calculated charges on Sn ion fall into two classes: 0.8~1.1 and ~1.5. Given that the charge transfer is generally underestimated by the Bader analysis, the above values are expected to correspond to Sn^{2+} and Sn^{4+}.

COHP analysis is essential to assess the bonding character between Sn and anions. We follow the conventional denotation of bonding (positive values) and antibonding (negative values) states and present –COHP in Fig. S11. It is noted that for Sn_2S_3 and $SnBr_2$, the Sn-*s* and S/Br-*p* orbitals overlap in the energy range of −7 ~ −5 eV where the –COHP is positive, indicating bonding interaction between Sn and anions. A relatively strong orbital overlap is also observed near the valence band maximum, which presents antibonding character. Hence, there appears to be a strong coupling between the Sn *s* and anion *p* states. This conclusion is also substantiated by the charge density between −7 and −5 eV shown in Fig. S12.

Electron localization function (ELF) is a measure of the conditional probability of finding an electron in the neighborhood of another electron with the same spin [6]. The extent of spatial localization of electrons can be estimated using this approach. That is

to say, the electron pair probability in multi-electronic systems can be rigorously mapped and quantified. ELF is calculated as follows:

$$\mathrm{ELF} = \frac{1}{1+(D/D^0)^2} \tag{S5}$$

$$D = \frac{1}{2}\sum_i^{\mathrm{occ}}|\nabla\varphi_i|^2 - \frac{|\nabla\rho|^2}{8\rho} \tag{S6}$$

$$D^0 = \frac{3}{10}(3\pi^2)^{5/3}\rho^{5/3} \tag{S7}$$

where φ_i denotes spin orbital and ρ is the electron spin density. D^0 is the counterpart of D for non-interaction homogenous electron gas. ELF varies within the range of [0,1]. A higher ELF value corresponds to a more localized distribution of electrons, generally indicating that there is covalent bonding, a lone electron pair or the inner shell of an atom [6-8].

Cation connection motifs and cationic percolation network/chain

Except for the cases where cations are directly connected through metallic bonding, the connection of cations in a compound can be described by the linkage between neighboring cation-centered coordination polyhedra. As a measure of cation connectivity, the connection motifs of cation-centered coordination polyhedra can be used to highlight the topological proximity between two cations, which does not rely on their interatomic distances. If both cations are linked by three or more anions, we say that both coordination polyhedra are face-sharing. When there are double and single linkages, they are defined as edge-sharing and vertex-sharing, respectively. In Fig. S13, we show the ratio of different cation connection motifs for LDMs, as compared with bulk compounds that cannot be partitioned into low-dimensional structural blocks.

We propose a 'rule-of-thumb' for estimating the degree of connectivity between neighboring cation-centered coordination polyhedra ('neighboring' means that they share common atoms): face/edge-sharing linkages of coordination polyhedra are defined as intimate connection, and vertex-sharing linkage is regarded as disconnected. While heuristic, this can serve the purpose for gauging the topological connectivity between two cations with the use of the structure graphs of the compounds. The identification protocol of cation percolation network/chain is nearly the same as the approach of computational exfoliation of bulk materials into LDMs. They differ in that only cation connectivity is involved in identifying the percolation network/chain, while for the construction of structure graphs, the connectivity of all atoms is taken into consideration. Both of the approaches are based on the topological information and can be leveraged for basic classification of different compounds. Figure S14 depicts several 2D structures that are characterized by such percolation network, while in Fig. S15, we present 2D structures without a cationic percolation network. The percentages of 2D

and 1D compounds with and without the cationic percolation network/chain are shown in Fig. S16.

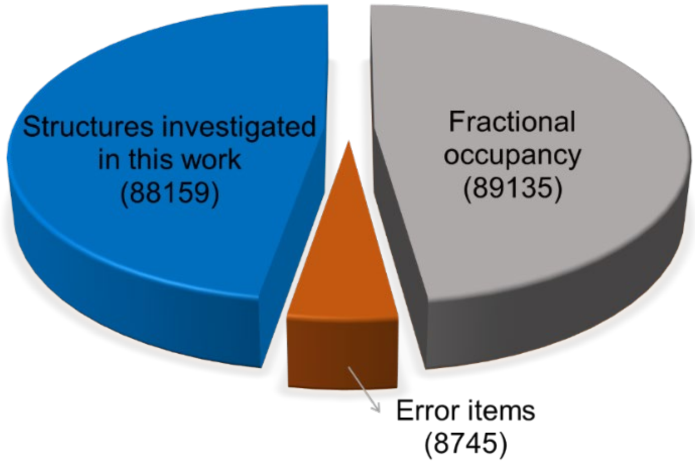

Figure S1. Database of bulk compounds from ICSD.

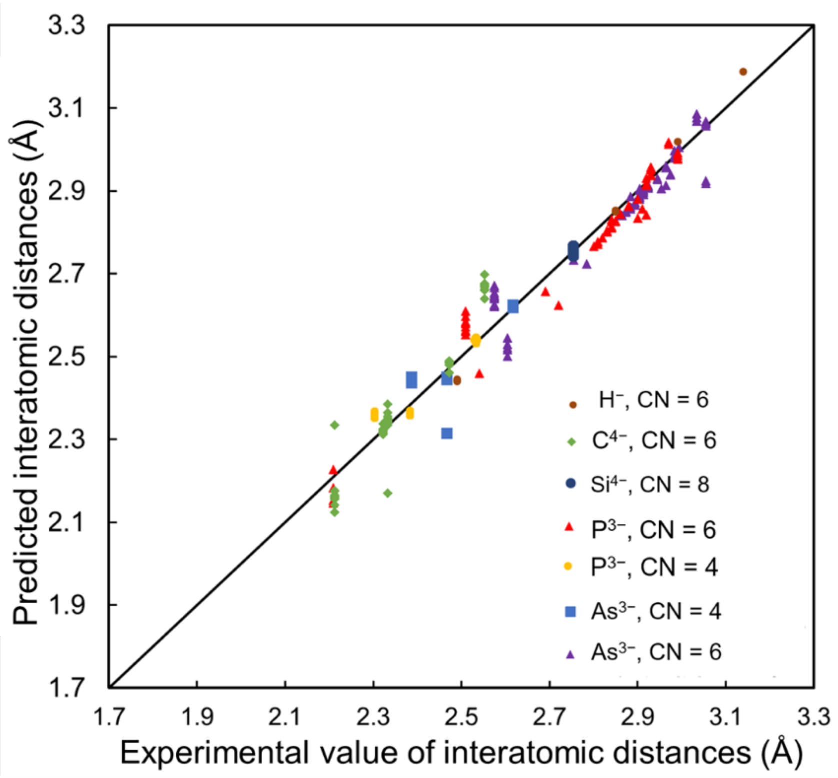

Figure S2. Comparison between the predicted and experimental values of interatomic distances for atom pairs containing H, C, Si, P and As anions with different coordination number (CN).

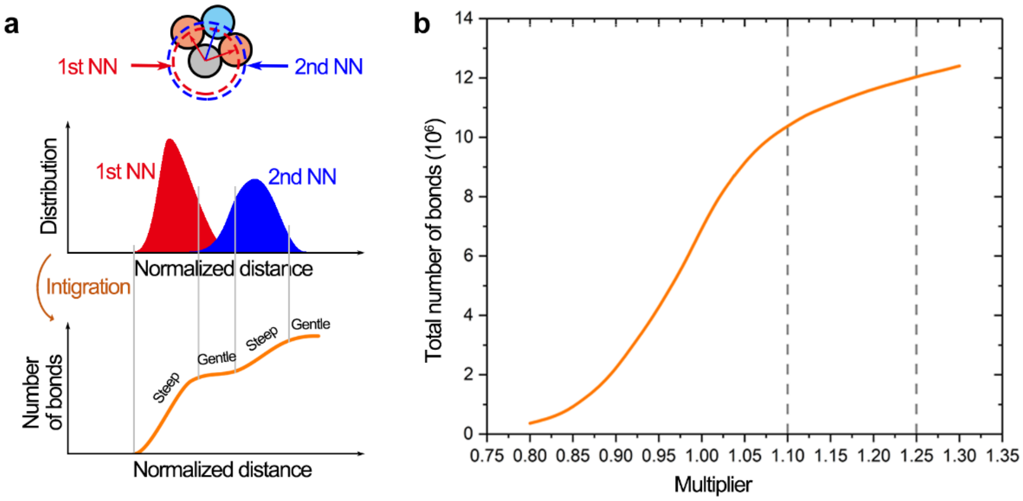

Figure S3. Justification of the chosen value of the multiplier (1.15). (a) Schematic illustration showing the distribution function of the interatomic distances normalized by atomic radii, and its integration, i.e., the total number of bonds in the whole collection of bulk compounds. (b) The total number of bonds for all bulk compounds as a function of the selected multiplier for identifying bond connections.

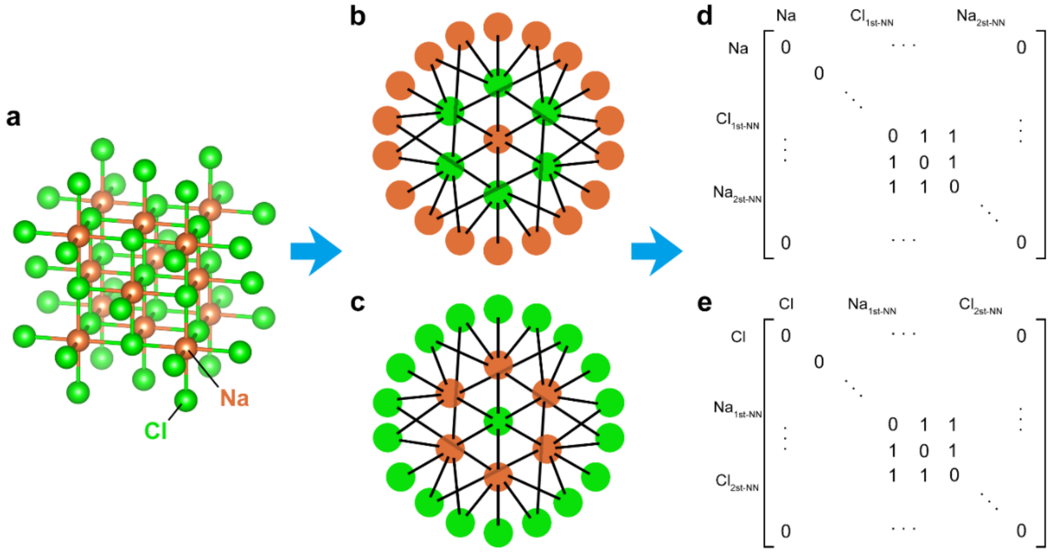

Figure S4. Construction of structure graph for NaCl. (a) Structure of NaCl. (b) Na-centered and (c) Cl-centered subgraphs. Adjacency matrices of the (d) Na-centered and (e) Cl-centered subgraphs.

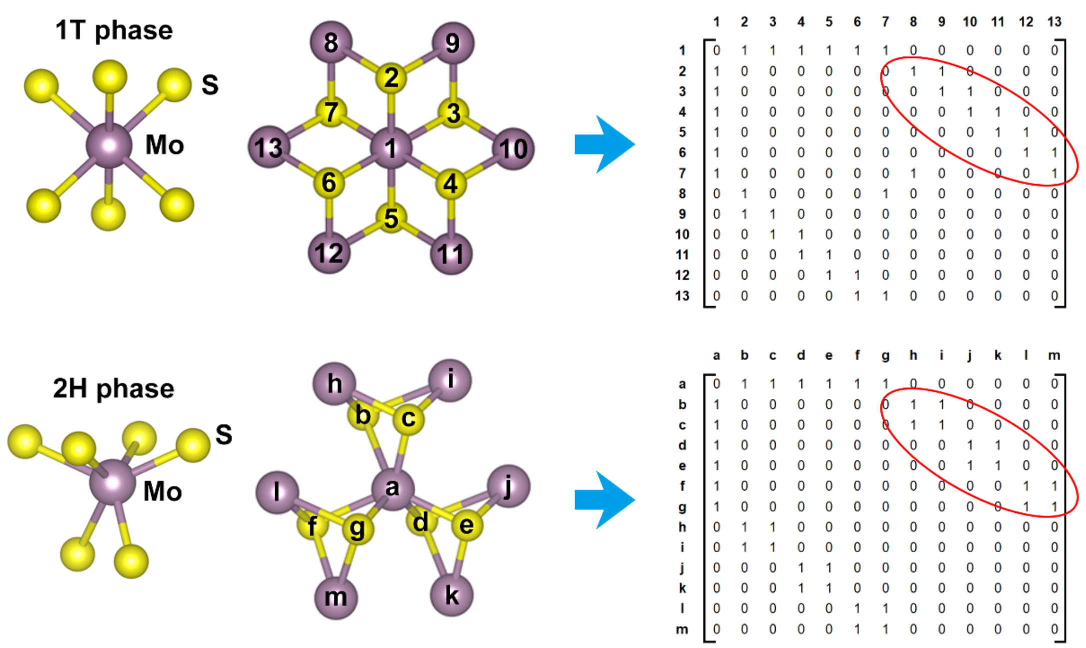

Figure S5. The 1st and 2nd nearest neighbors of 1T and 2H phases of MoS_2 monolayer, and their corresponding adjacency matrices for the subgraphs centered on Mo atom.

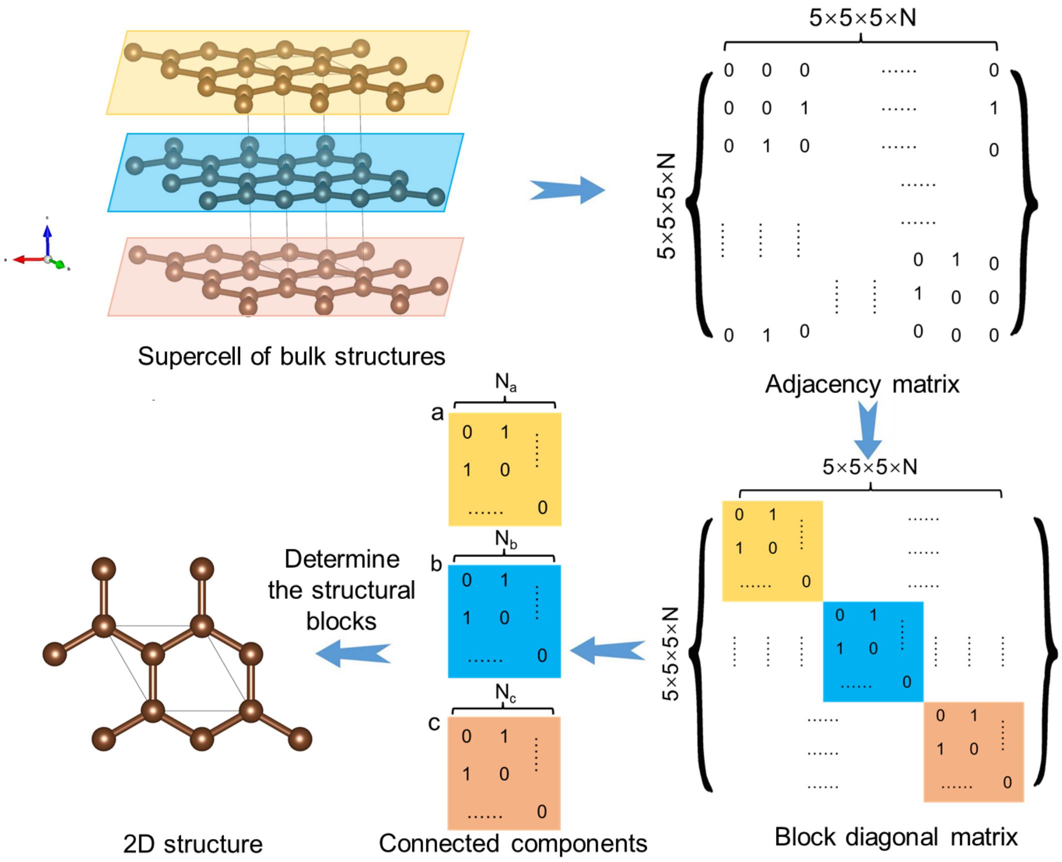

Figure S6. Identification of structural blocks using the structure graph of supercell. Based on the adjacency matrix, the structure of 2D graphene can be extracted from supercell structure graph of graphite.

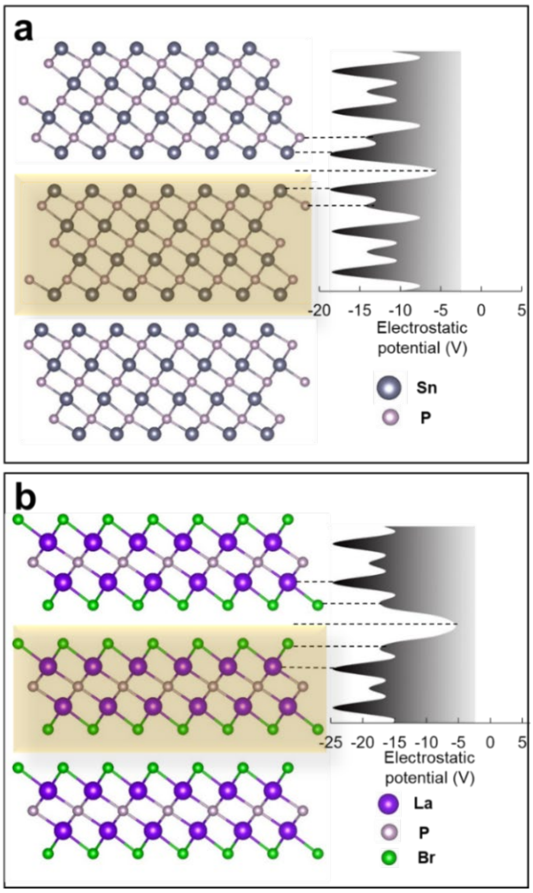

Figure S7. Structures of Sn_4P_3 and La_2PBr_2. Structural blocks of (a) Sn_4P_3 and (b) La_2PBr_2, and the corresponding plane-averaged electrostatic potentials along the layer normal.

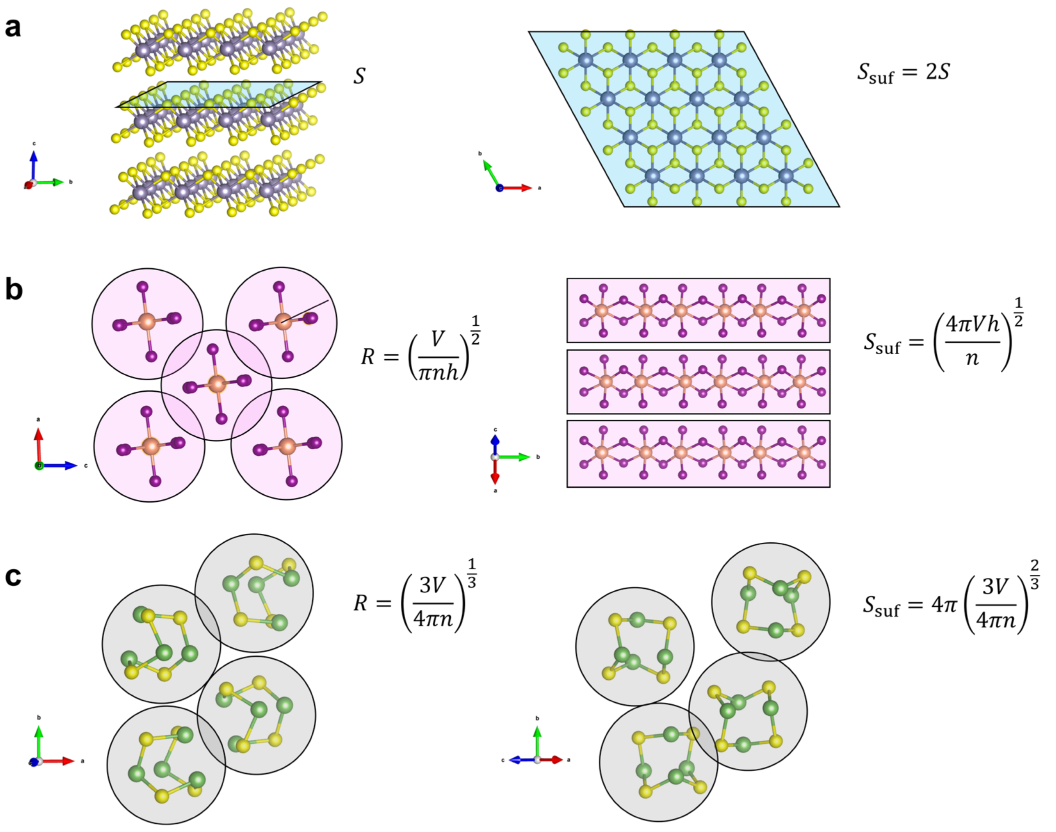

Figure S8. The effective surface area for 2D, 1D and 0D materials. (a) 2D atomic layers. (b) 1D atomic chains. (c) 0D atomic clusters.

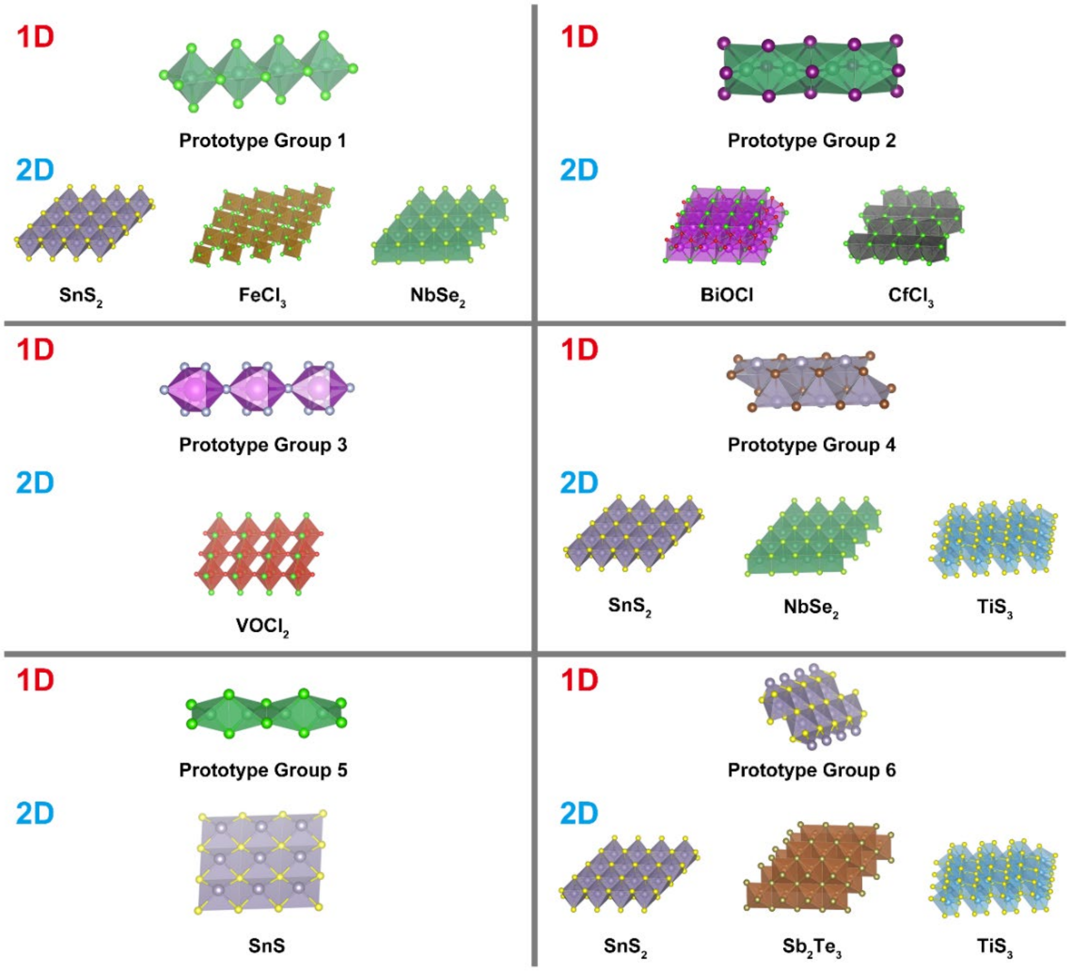

Figure S9. 2D structures that can be truncated into one of the six most popular 1D structural prototypes.

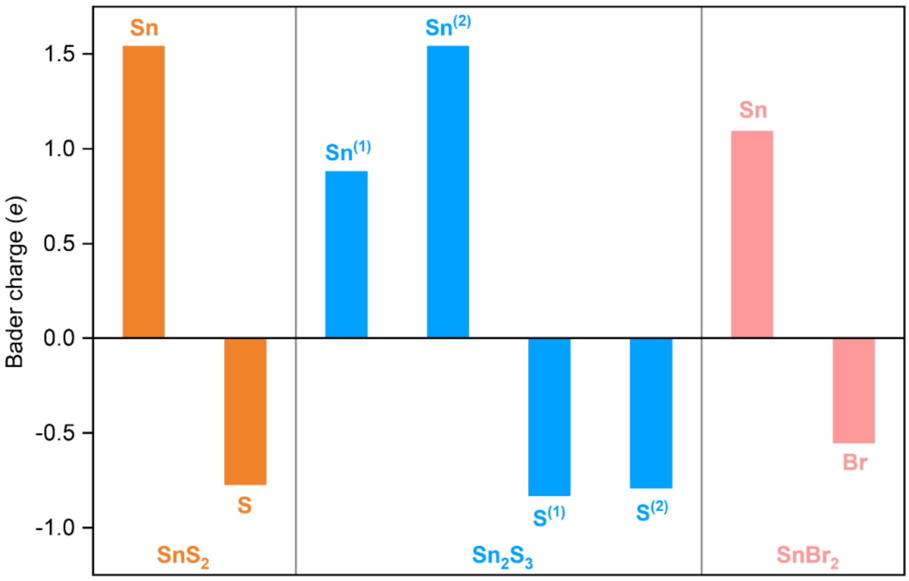

Figure S10. Bader charges of different ions in SnS_2, Sn_2S_3 and $SnBr_2$.

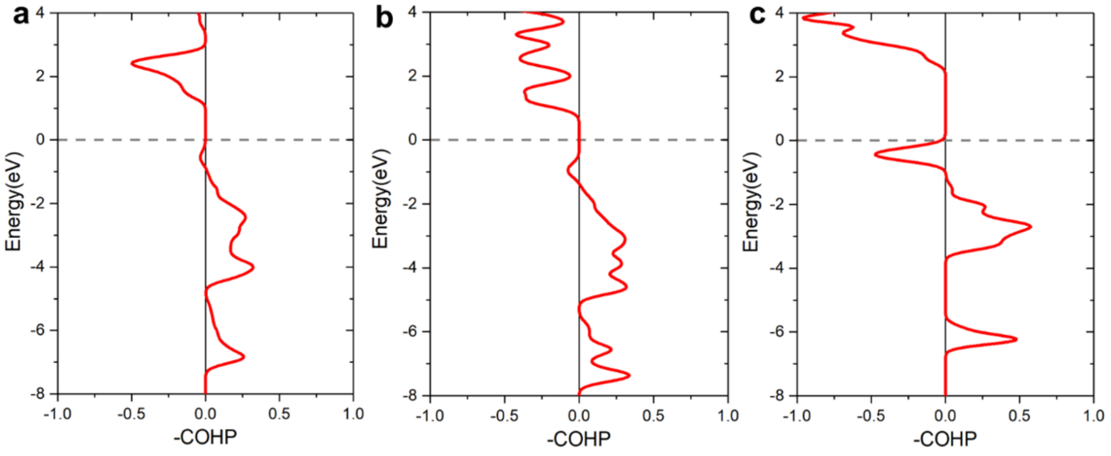

Figure S11. COHP for Sn-anion bonds. (a) Sn-S in SnS_2. (b) Sn-S in Sn_2S_3. (c) Sn-Br in $SnBr_2$. Energies are referenced to the Fermi level.

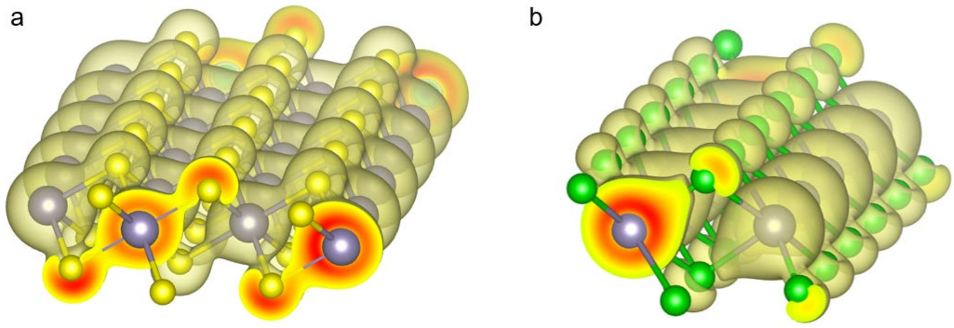

Figure S12. Charge density of states between −7 and −5 eV *vs.* the Fermi energy. (a) Sn_2S_3. (b) $SnBr_2$. Color code: Sn grey, S yellow, and Br green.

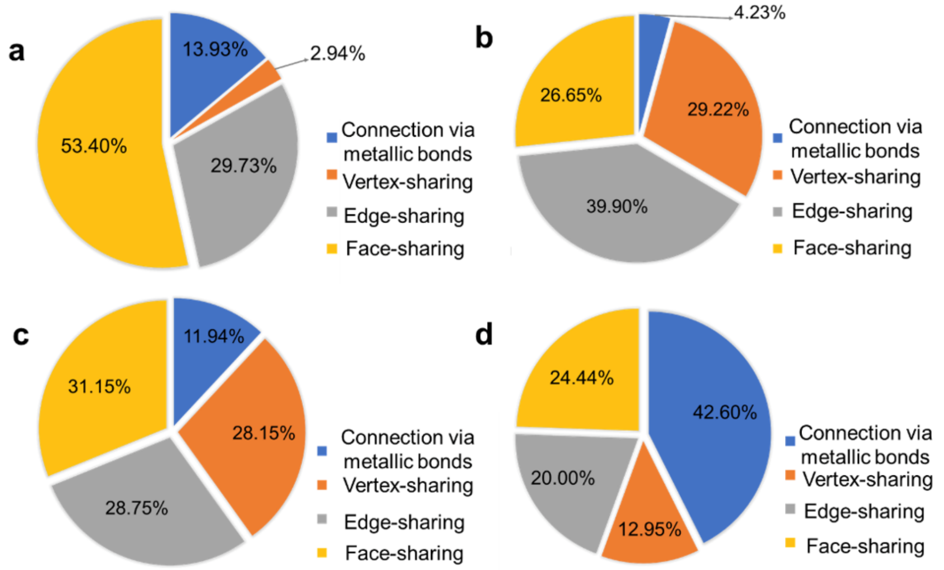

Figure S13. Statistics on the cation connection motifs. (a) Bulk compounds that cannot be partitioned into low-dimensional structural blocks. (b) 2D atomic layers. (c) 1D atomic chain. (d) 0D atomic clusters.

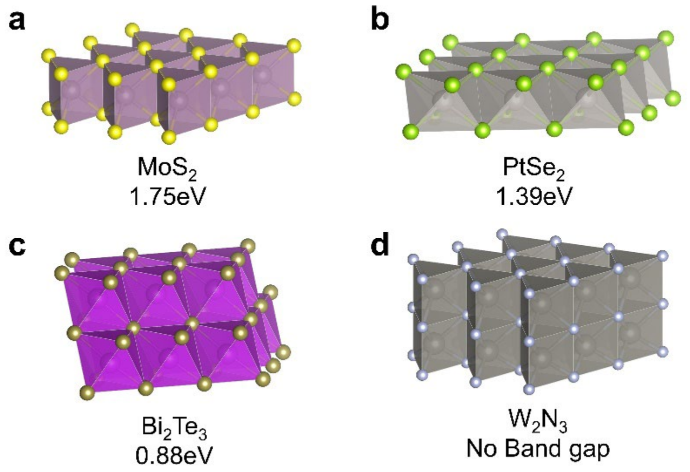

Figure S14. 2D structures with cationic percolation network, and their electronic band gaps. (a) MoS_2. (b) $PtSe_2$. (c) Bi_2Te_3. (d) W_2N_3.

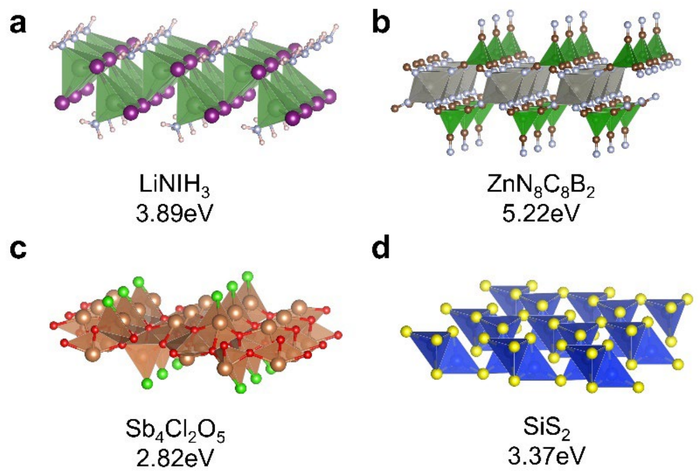

Figure S15. 2D structures without cationic percolation network, and their electronic band gaps. (a) $LiNIH_3$. (b) $ZnN_8C_8B_2$. (c) $Sb_4Cl_2O_5$. (d) SiS_2.

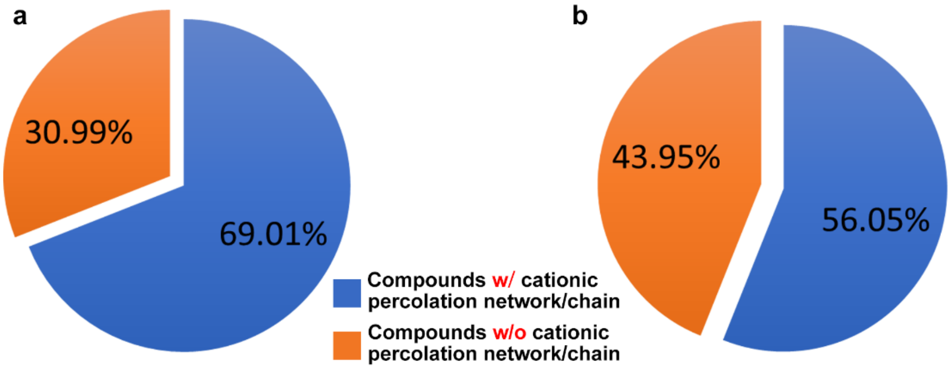

Figure S16. The percentages of compounds with and without cationic percolation network/chain. (a) 2D atomic layers. (b) 1D atomic chains.

Table S1. Covalent, metallic and ionic radii for all elements considered in this work.

Element	Covalent radius (Å)	Metallic radius (Å)	Ionic radius (Å)	Element	Covalent radius (Å)	Metallic radius (Å)	Ionic radius (Å)
H	0.37	/	1.33	Rh	1.25	1.35	0.81
Li	1.23	1.52	1.06	Pd	1.28	1.38	1.00
Be	0.89	1.13	0.59	Ag	1.34	1.44	1.42
B	0.83	/	0.41	Cd	1.41	1.49	1.45
C	0.77	/	1.47	In	1.50	1.63	1.06
N	0.70	/	1.32	Sn	1.40	1.41	0.95
O	0.66	/	1.28	Sb	1.41	1.41	0.94
F	0.64	/	1.19	Te	1.37	1.43	2.07
Na	1.57	1.54	1.53	I	1.33	/	2.06
Mg	1.36	1.60	1.03	Cs	2.35	2.61	2.02
Al	1.25	1.43	0.68	Ba	1.98	2.17	1.75
Si	1.17	/	2.04	La	1.69	1.88	1.50
P	1.10	/	1.80	Ce	1.64	1.83	1.48
S	1.04	/	1.70	Pr	1.65	1.83	1.32
Cl	0.99	/	1.67	Nd	1.64	1.82	1.49
K	2.03	2.28	1.78	Pm	/	1.81	1.28
Ca	1.74	1.97	1.40	Sm	1.66	1.80	1.46
Sc	1.44	1.61	1.01	Eu	1.85	2.04	1.49
Ti	1.32	1.45	1.00	Gd	1.61	1.80	1.25
V	1.22	1.32	0.93	Tb	1.59	1.78	1.24
Cr	1.17	1.25	0.94	Dy	1.56	1.77	1.33
Mn	1.17	1.24	1.10	Ho	1.58	1.77	1.26
Fe	1.17	1.24	1.06	Er	1.57	1.76	1.20
Co	1.16	1.25	1.04	Tm	1.56	1.75	1.23
Ni	1.15	1.25	0.83	Yb	1.70	1.94	1.28
Cu	1.17	1.28	0.91	Lu	1.56	1.73	1.17
Zn	1.25	1.33	1.04	Hf	1.44	1.56	0.97
Ga	1.25	1.22	0.76	Ta	1.34	1.43	0.88
Ge	1.22	1.23	0.87	W	1.30	1.37	0.80
As	1.21	1.25	1.86	Re	1.28	1.37	0.77
Se	1.17	/	1.84	Os	1.26	1.34	0.77
Br	1.14	/	1.82	Ir	1.26	1.36	0.82
Rb	2.16	2.48	1.97	Pt	1.29	1.38	0.94
Sr	1.92	2.15	1.58	Au	1.34	1.44	1.51
Y	1.62	1.81	1.22	Hg	1.44	1.60	1.33
Zr	1.45	1.60	1.03	Tl	1.55	1.70	1.84
Nb	1.34	1.43	0.93	Pb	1.54	1.75	1.63
Mo	1.29	1.36	0.87	Po	1.53	1.67	1.22
Tc	/	1.36	0.79	Bi	1.52	1.55	1.31
Ru	1.24	1.33	0.82				

Table S2. The formula, structure, structure unit, and configuration after MD simulation of the representative 1D materials.

Proto-type	Formula	Structure	Structure unit	Configuration after MD simulation	Element
1	$NbCl_4$	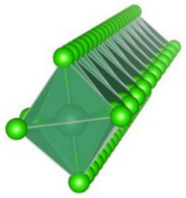	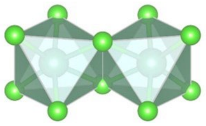	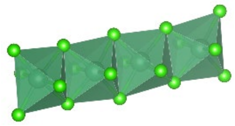	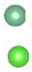
1	$OsCl_4$	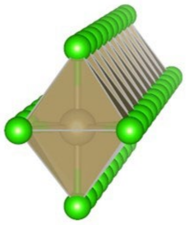	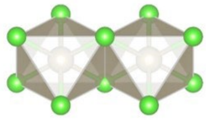	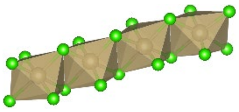	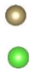
1	WCl_4	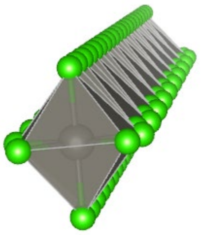	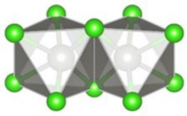	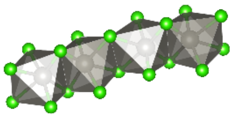	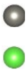
1	NbI_4	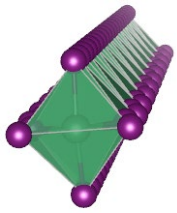	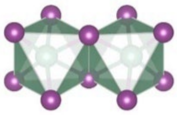	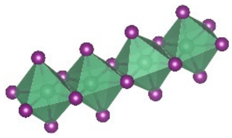	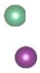
1	$NbBr_4$	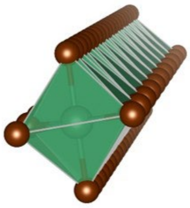	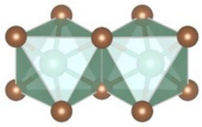	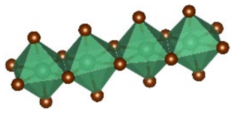	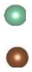
1	$TaCl_4$	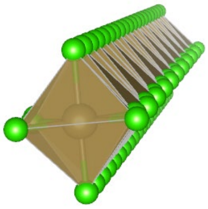	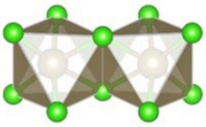	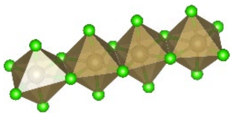	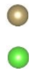

1	WBr_4	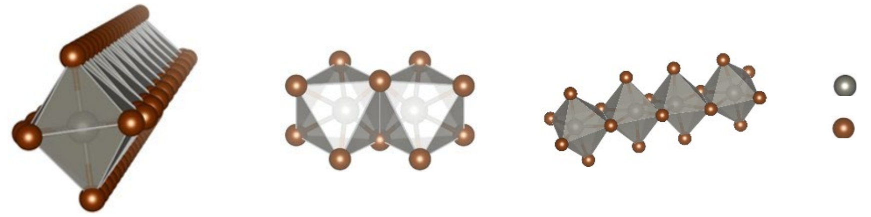
1	$ReSCl_3$	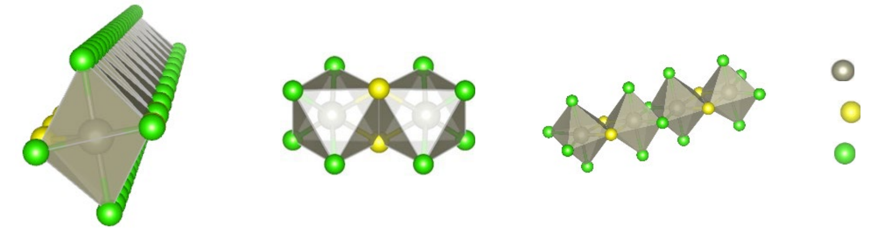
1	TaI_2Cl_2	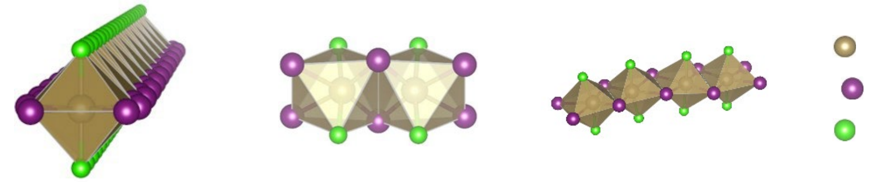
1	NbI_2Cl_2	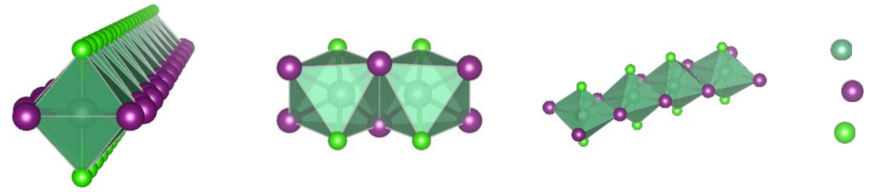
1	PtI_4	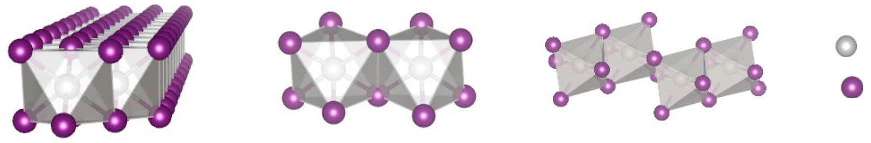
1	$TcBr_4$	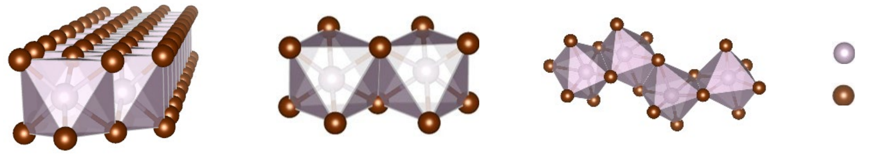
1	$ZrCl_4$	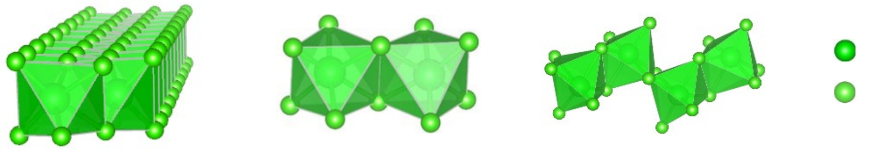
1	$TcCl_4$	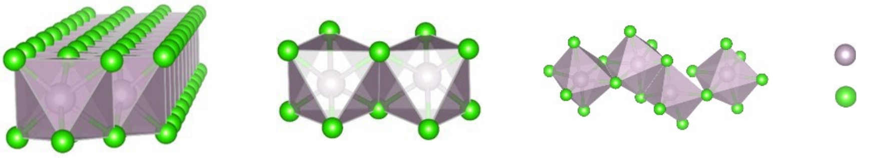
1	$HfCl_4$	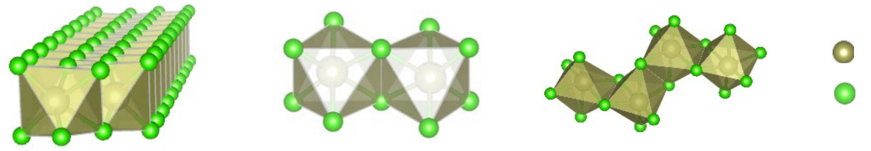

1	$OsBr_4$	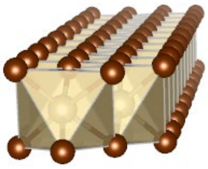	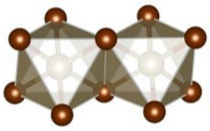	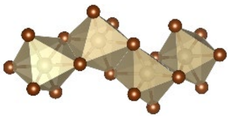	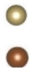
1	ReO_3F	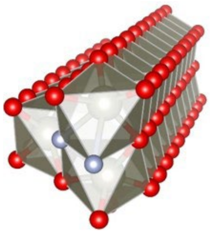	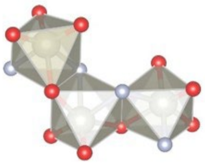	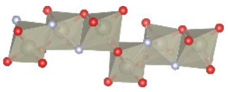	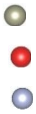
1	HfI_4	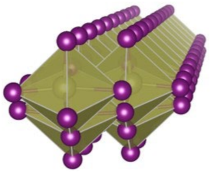	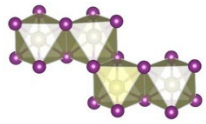	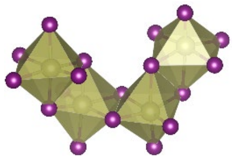	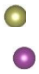
1	ZrI_4	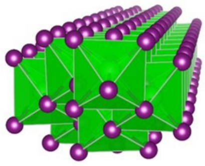	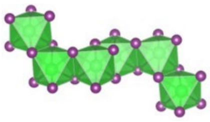	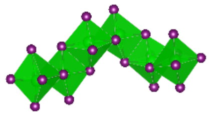	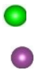
2	NbI_3	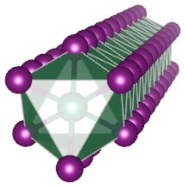	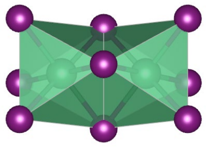	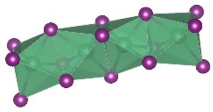	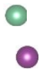
2	$ZrBr_3$	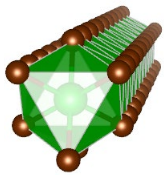	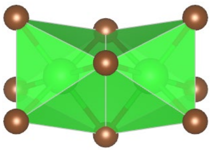	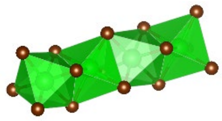	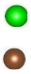
2	TiI_3	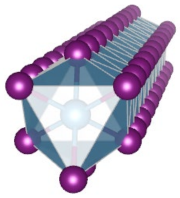	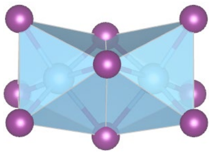	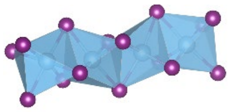	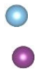
2	$RuCl_3$	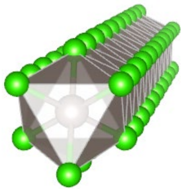	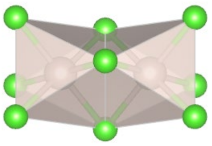	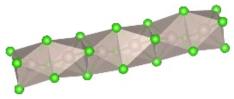	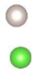

2	$ZrCl_3$	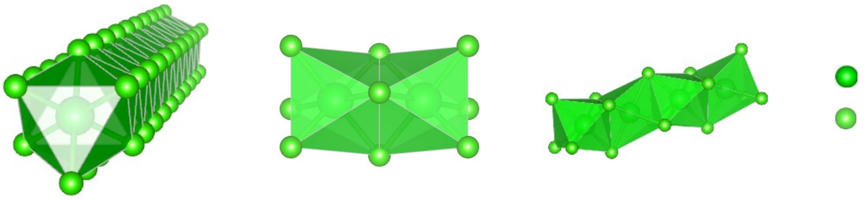
2	ZrI_3	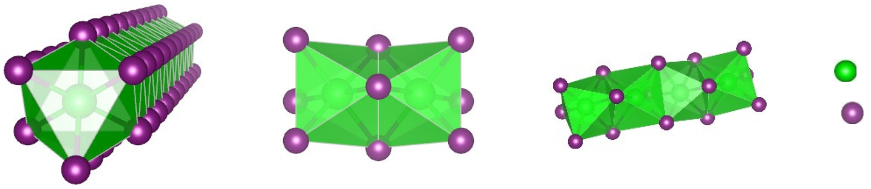
2	HfI_3	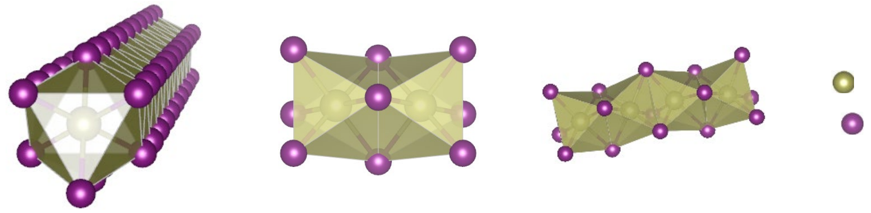
2	MoI_3	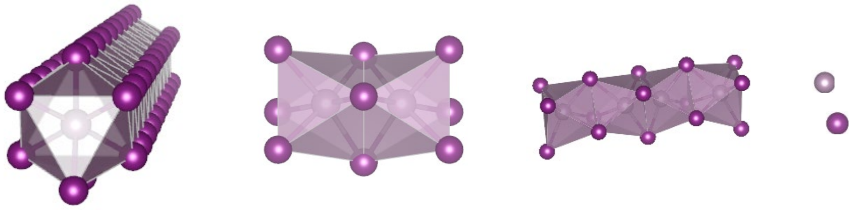
2	$TcBr_3$	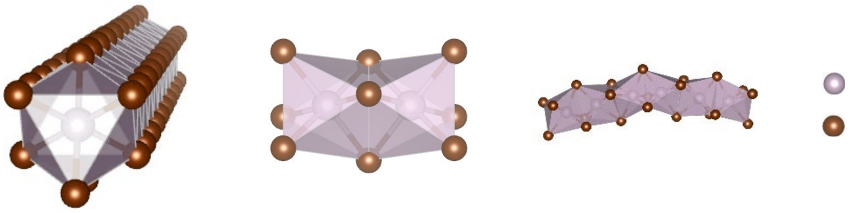
2	$TiCl_3$	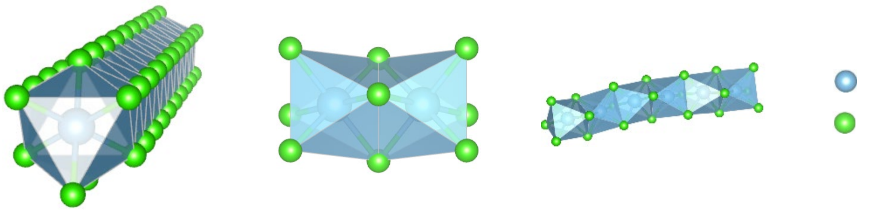
2	$RuBr_3$	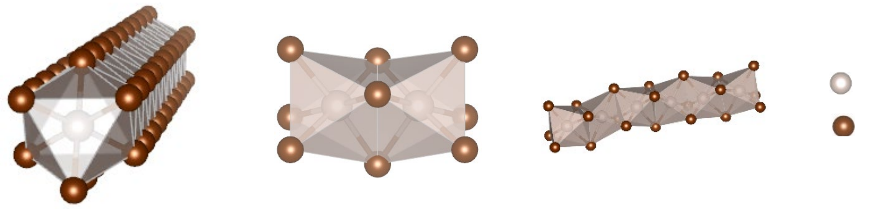

2	$MoBr_3$	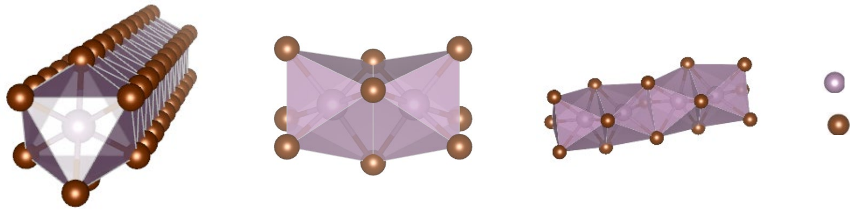
3	BiF_5	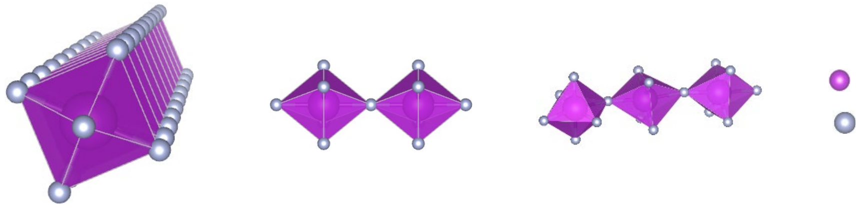
3	$WOCl_4$	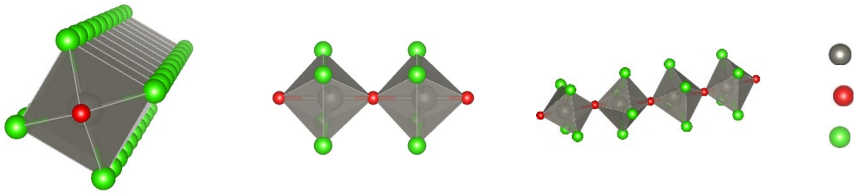
3	$WOBr_4$	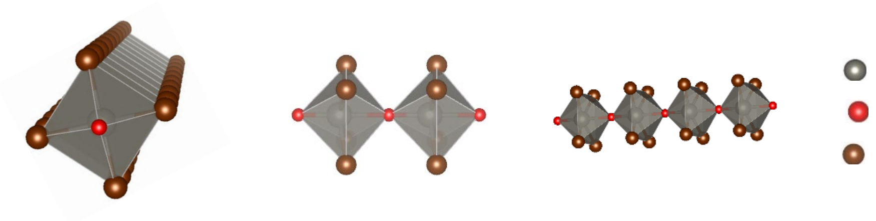
3	NbI_5	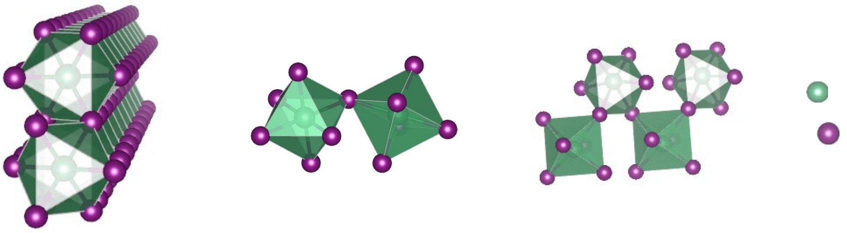
3	CrF_5	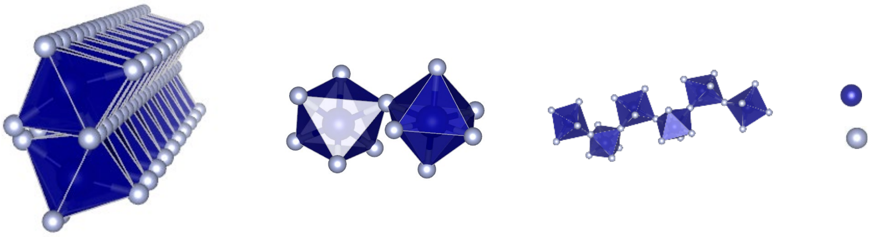
3	$NbSbF_{10}$	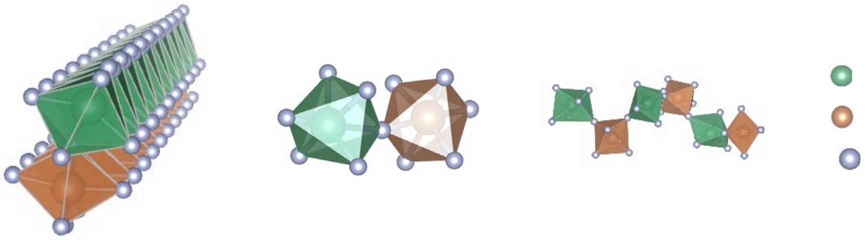
3	$Os_2O_4F_6$	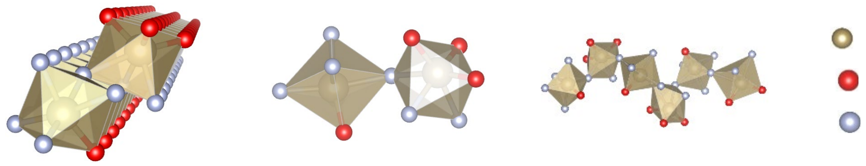

3	$OsOF_4$	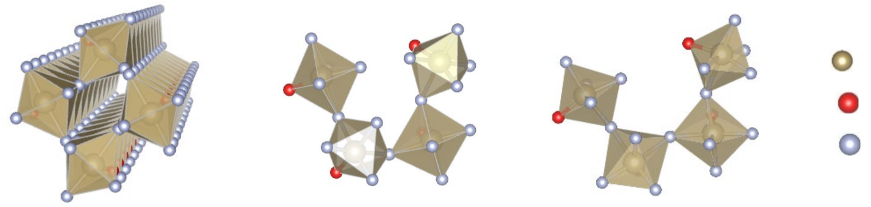
3	$CrSbF_{10}$	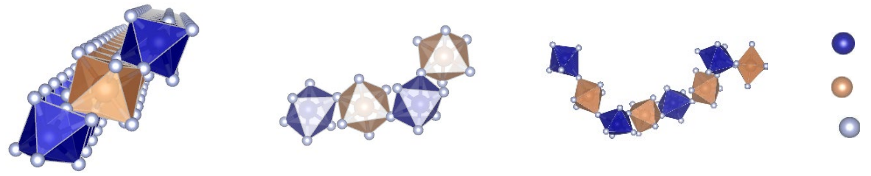
3	$SbReO_2F_8$	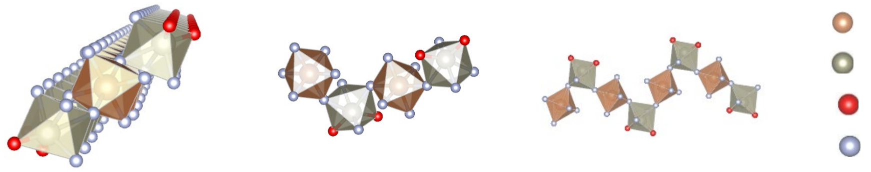
3	$MoSbOF_9$	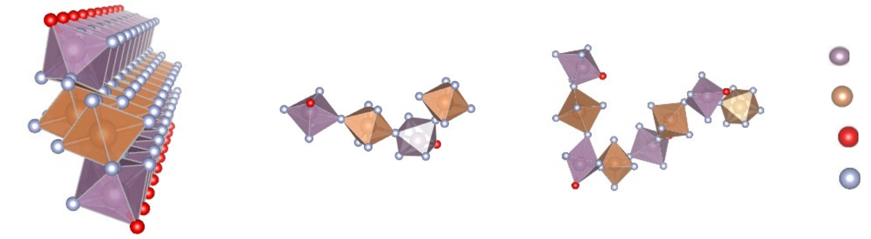
4	$SnBr_2$	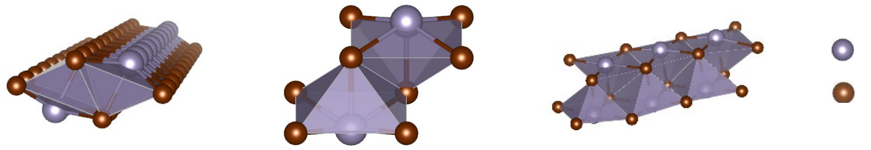
4	SnICl	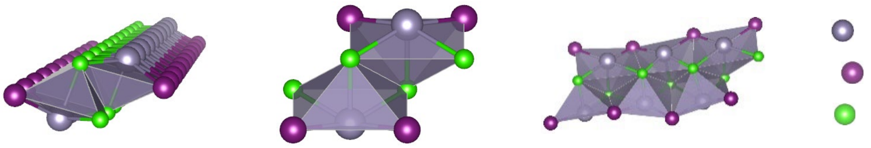
4	SbSBr	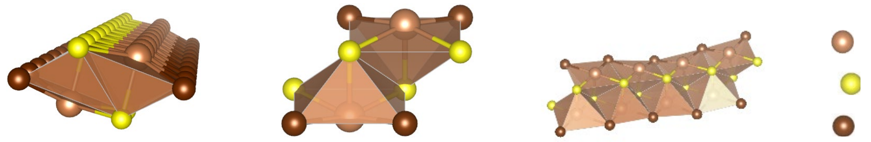
4	SbSI	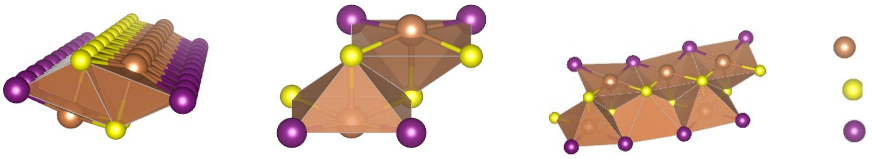
4	SbSeI	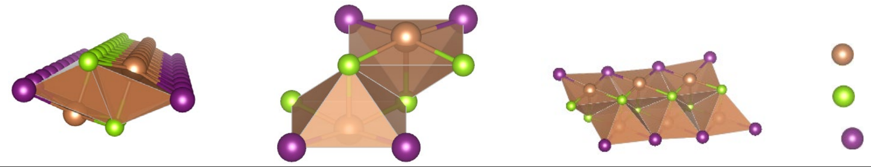

4	SbTeI	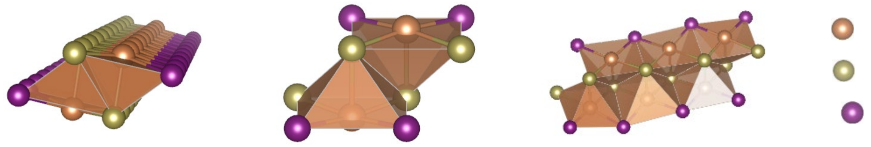
4	BiSI	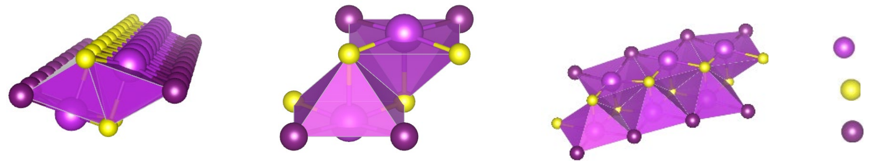
4	BiSeBr	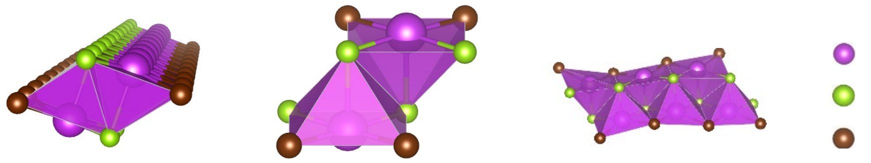
5	$BeCl_2$	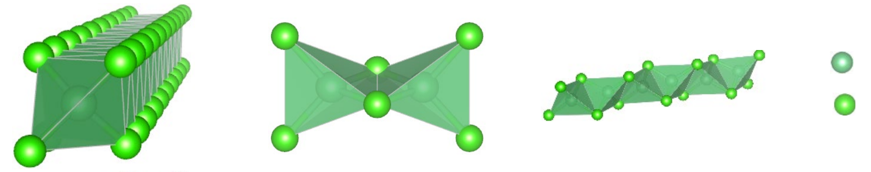
5	$BeBr_2$	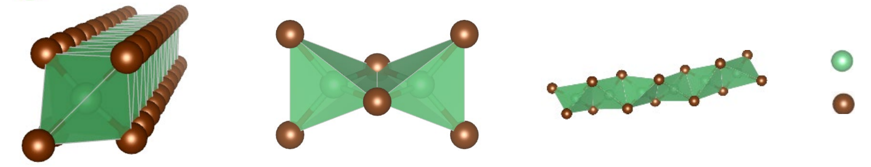
5	BeI_2	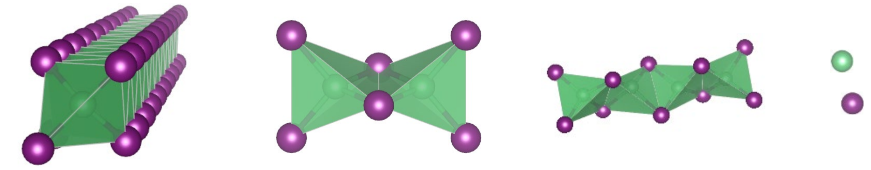
5	SiO_2	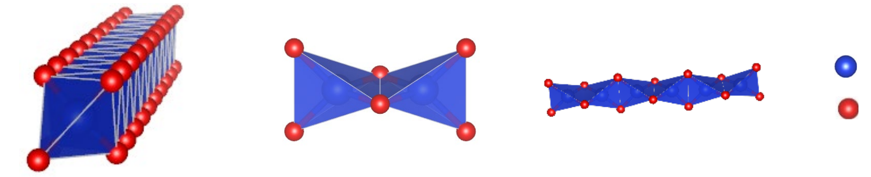
5	SiS_2	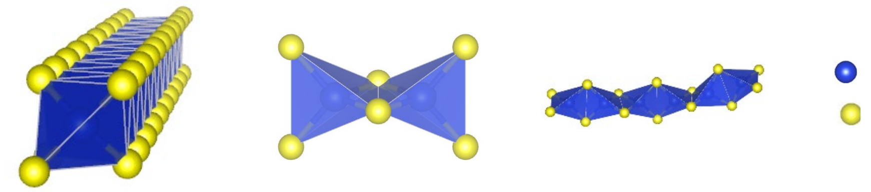
5	$SiSe_2$	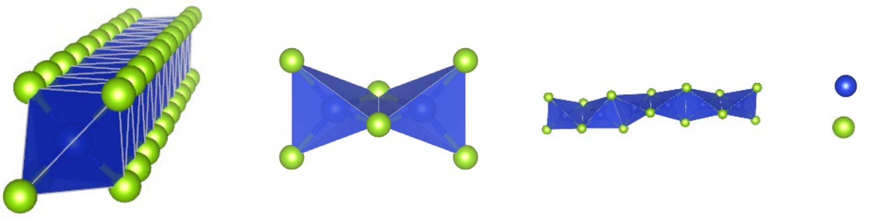

5	$AlPS_4$	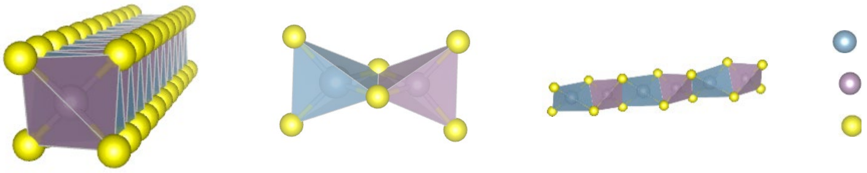
5	BPS_4	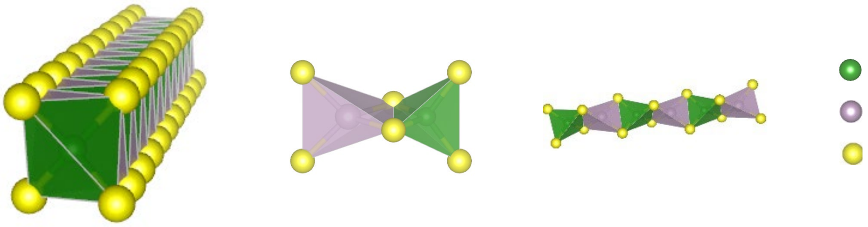
6	Sn_2S_3	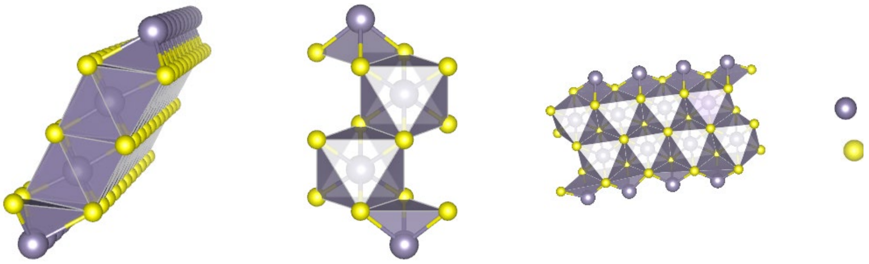
6	$GeTiS_3$	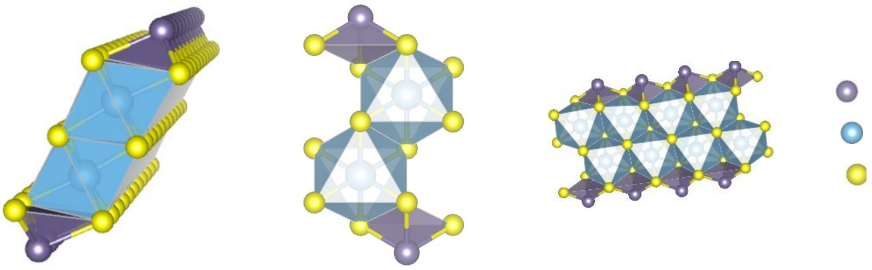
6	$SbInS_3$	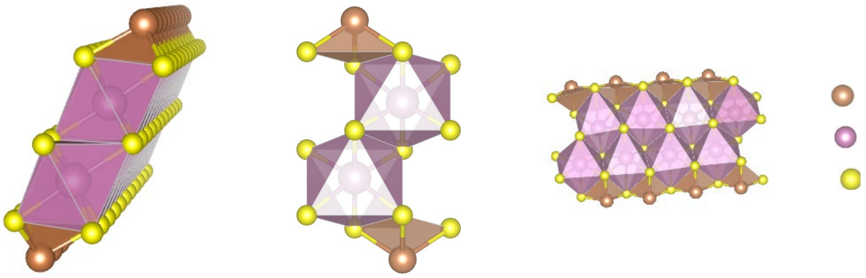
6	$HfSnS_3$	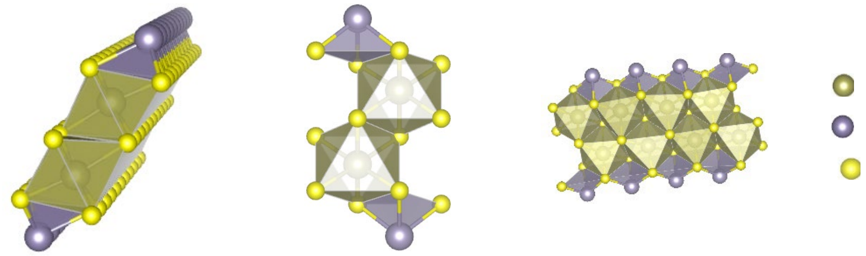

6	$ZrSnS_3$	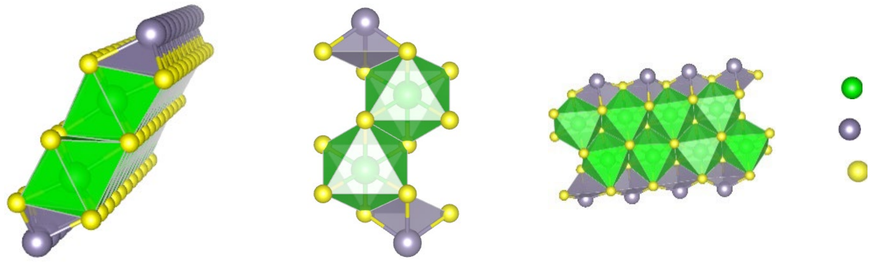
6	As_2Te_3	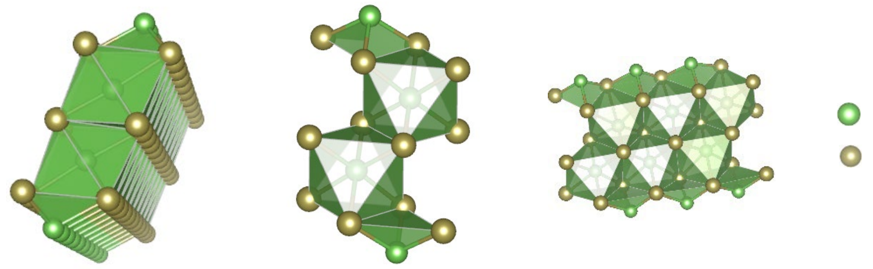
6	As_2Se_3	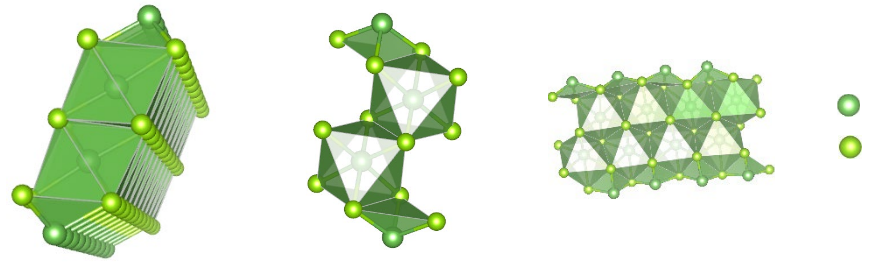
7	$CoAl_2Cl_8$	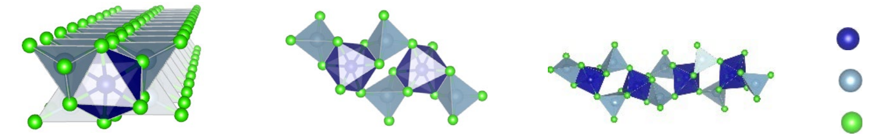
7	$TiAl_2Cl_8$	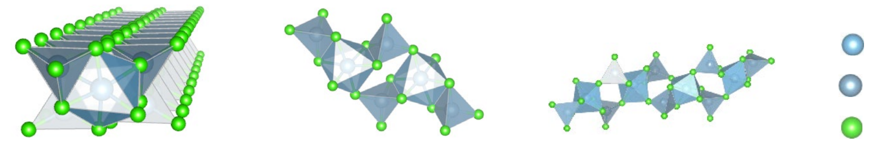
7	VAl_2Cl_8	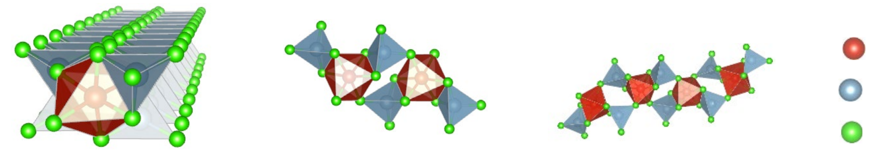
7	$MgAl_2Cl_8$	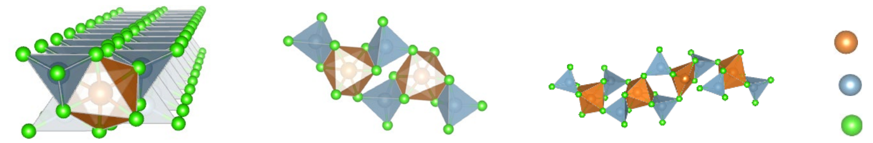
7	$CrAl_2Cl_8$	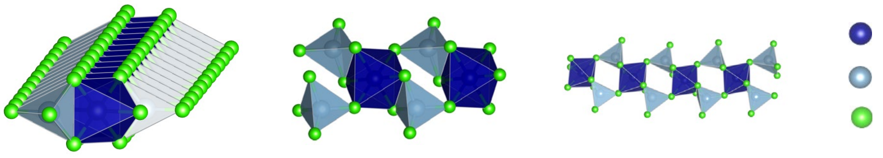

8	$NbSeCl_3$	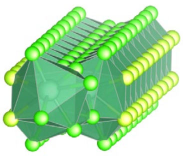	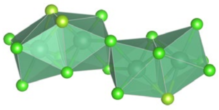	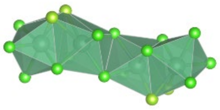	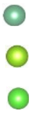
8	$NbSeBr_3$	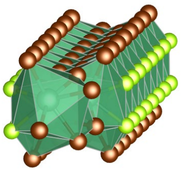	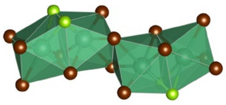	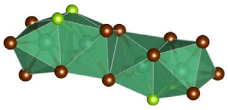	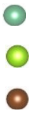
8	$NbSeI_3$	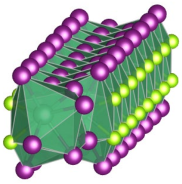	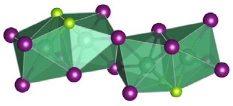	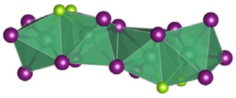	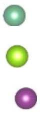
8	$NbTeBr_3$	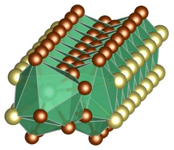	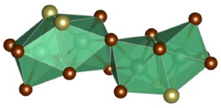	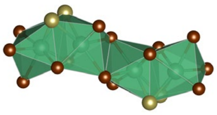	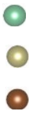
8	$NbTeI_3$	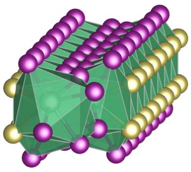	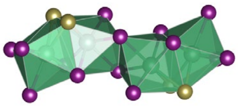	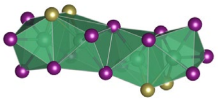	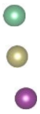
9	CrF_4	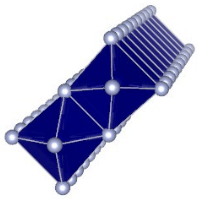	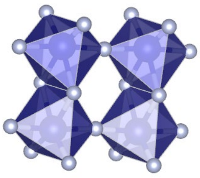	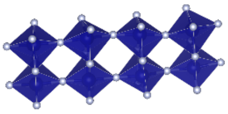	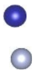
9	$NbOCl_3$	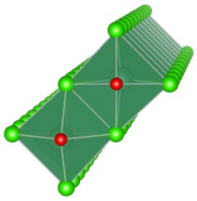	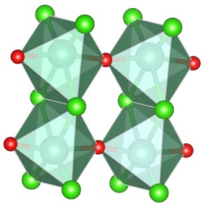	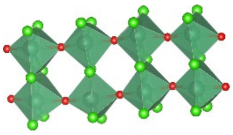	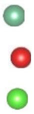
9	$WOCl_3$	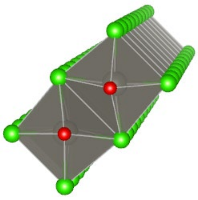	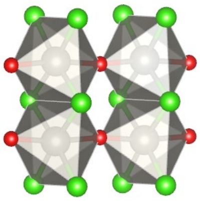	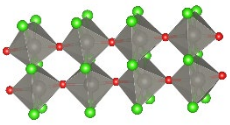	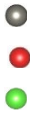

9	$NbOBr_3$	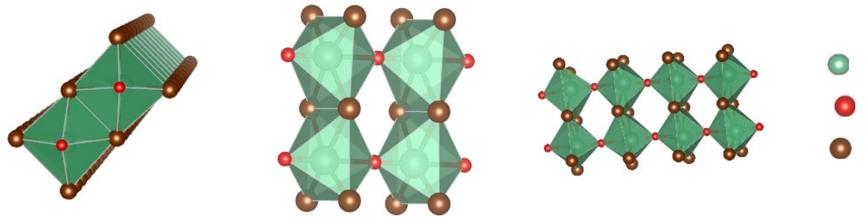
9	WOI_3	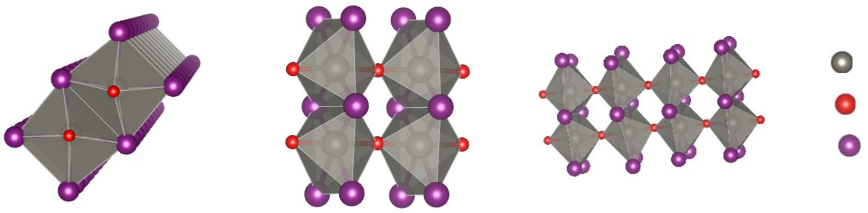
10	PNF_2	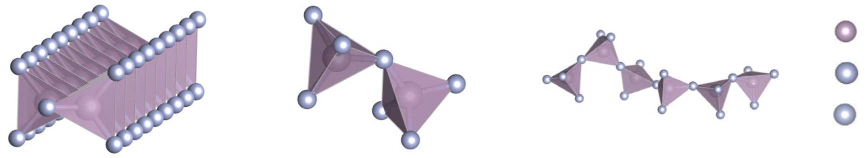
10	SO_3	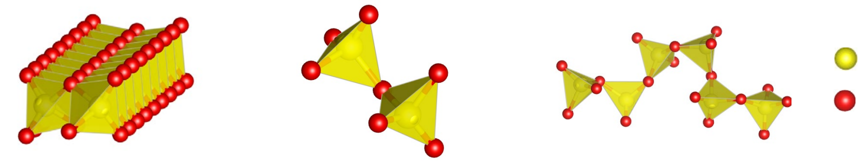
10	$GaSbCl_6$	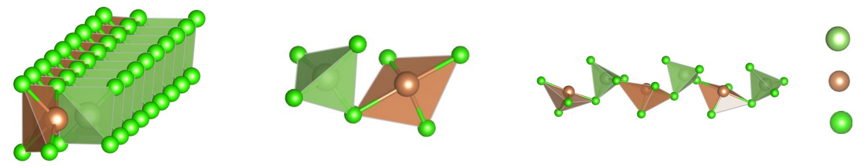
10	$TeOF_2$	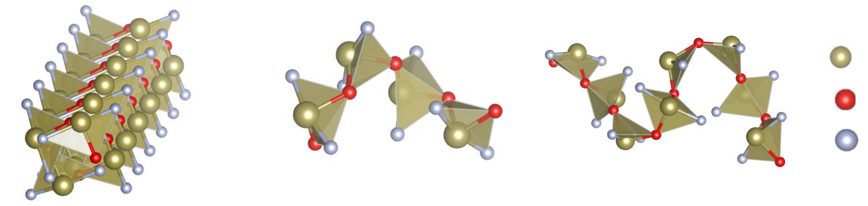
10	CrO_3	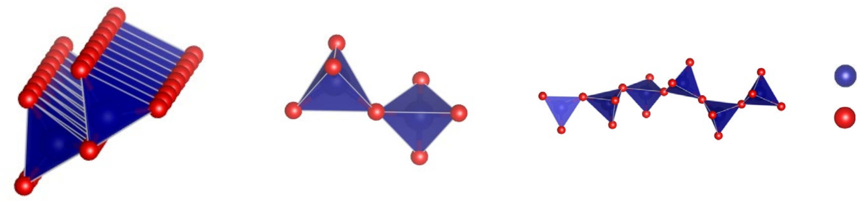

Table S3. The formula, ICSD number (parent bulk crystal) and exfoliation energy of the representative 1D materials.

Proto-type	Formula	ICSD	Exfoliation energy (meV/Å^2)	Formula	ICSD	Exfoliation energy (meV/Å^2)
1	$NbCl_4$	1010	5.34	HfI_4	200826	5.90
	$OsCl_4$	1165	5.44	$NbBr_4$	239640	6.18
	ZrI_4	8068	6.07	$TcCl_4$	260163	5.77
	PtI_4	15173	8.78	$HfCl_4$	402054	4.67
	NbI_4	23916	6.96	$TaCl_4$	402406	5.60
	$ZrCl_4$	26049	4.54	NbI_2Cl_2	407489	6.75
	$TcBr_4$	26055	4.77	ReO_3F	415418	9.52
	$OsBr_4$	61042	6.61	WBr_4	423461	6.96
	TaI_2Cl_2	69688	6.58	$ReSCl_3$	429458	6.80
	WCl_4	165263	6.11			
2	$RuCl_3$	22090	5.80	NbI_3	109145	6.93
	$ZrCl_3$	23163	5.39	$ZrBr_3$	165302	5.89
	ZrI_3	23946	5.89	TiI_3	173784	6.21
	HfI_3	23947	6.31	MoI_3	242166	6.47
	$TiCl_3$	26069	5.03	$TcBr_3$	260162	6.04
	$RuBr_3$	28119	6.82	$MoBr_3$	413690	5.97
3	$NbSbF_{10}$	16095	6.78	$Os_2O_4F_6$	240330	7.68
	BiF_5	25023	4.99	$SbReO_2F_8$	280248	6.86
	NbI_5	25503	6.88	$OsOF_4$	417245	8.09
	$WOCl_4$	25519	5.04	CrF_5	419661	5.42
	$WOBr_4$	49547	6.56	$CrSbF_{10}$	419662	7.13
	$MoSbOF_9$	201410	8.78			
4	SnICl	23262	8.15	SbSeI	31292	10.65
	SbSBr	25571	10.55	SbTeI	31355	10.81
	SbSI	25572	10.54	BiSeBr	76649	11.32
	BiSI	25575	11.28	$SnBr_2$	411177	7.64
5	$AlPS_4$	15910	4.75	SiS_2	26858	4.98
	$SiSe_2$	24592	5.80	$BeCl_2$	31696	3.77
	BPS_4	24618	5.07	$BeBr_2$	92584	4.30
	SiO_2	25632	6.03	BeI_2	92585	4.62
6	Sn_2S_3	15338	12.48	$GeTiS_3$	263128	11.98
	As_2Te_3	18208	13.51	$SbInS_3$	300207	18.59
	$HfSnS_3$	65667	13.97	As_2Se_3	611373	12.65
	$ZrSnS_3$	73711	14.09			
7	$CoAl_2Cl_8$	22143	4.49	VAl_2Cl_8	415951	4.63
	$TiAl_2Cl_8$	39565	4.60	$CrAl_2Cl_8$	416836	4.80
	$MgAl_2Cl_8$	62046	4.28			

8	$NbSeBr_3$	35375	7.41	$NbSeCl_3$	172519	7.22
	$NbTeBr_3$	35376	8.36	$NbSeI_3$	410743	7.56
	$NbTeI_3$	35377	8.49			
9	$NbOCl_3$	26471	5.07	$WOCl_3$	416393	5.08
	$WOBr_3$	65183	7.43	$NbOBr_3$	418089	6.50
	CrF_4	78778	6.08			
10	PNF_2	9684	4.93	$TeOF_2$	88415	17.61
	SO_3	24723	10.00	CrO_3	109366	12.81
	$GaSbCl_6$	24786	3.97			

Table S4. The formula, structure, and ICSD number (parent bulk crystal) of the representative 0D materials.

Prototype	Formula	Structure	Element	ICSD
1	Rh_4F_{20}	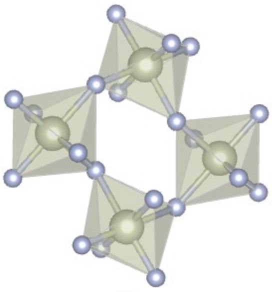	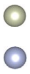	10173
1	Ru_4F_{20}	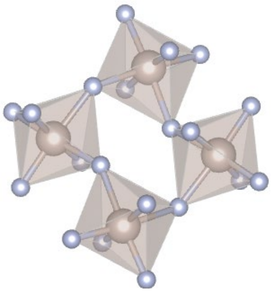	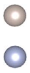	165397
1	$Sb_4Cl_{12}F_8$	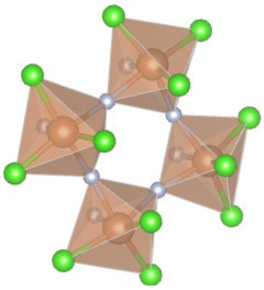	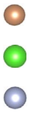	200039
1	$Os_4F_{12}O_8$	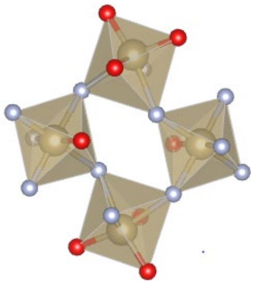	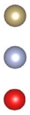	240331
1	$Nb_4Cl_{16}F_4$	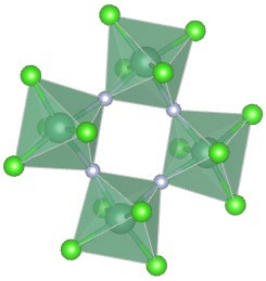	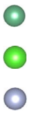	26155

1	$Ta_4Cl_{16}F_4$	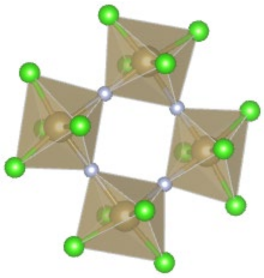	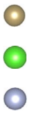	27413
1	$Sb_4Cl_{16}F_4$	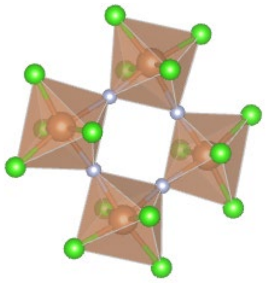	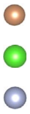	30629
2	Re_2Cl_{10}	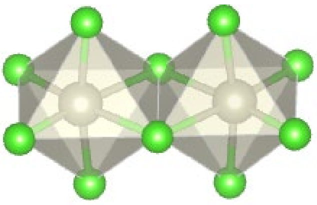	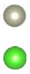	22139
2	Nb_2Br_{10}	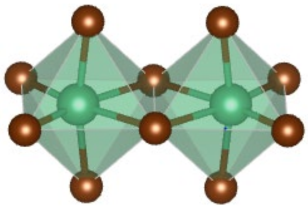	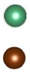	409917
2	Sb_2Cl_{10}	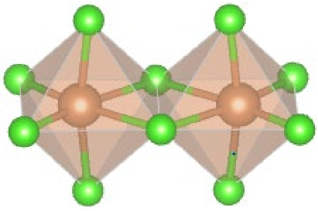	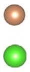	412110
2	Nb_2Cl_{10}	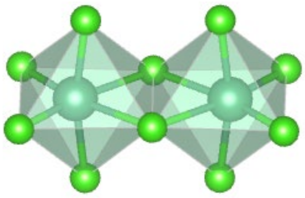	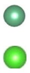	66537
2	Mo_2Cl_{10}	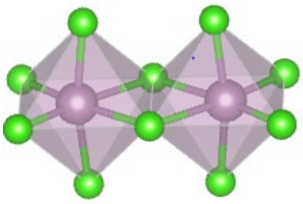	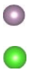	84622

2	$Re_2Cl_6O_4$	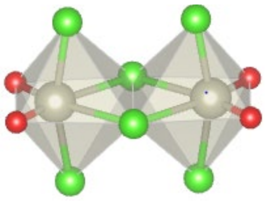	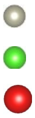	416056
3	P_4O_{10}	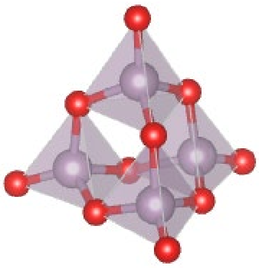	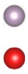	16610
3	P_4S_{10}	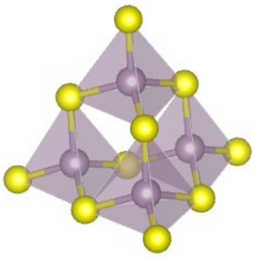	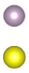	174009
3	$P_4S_4O_6$	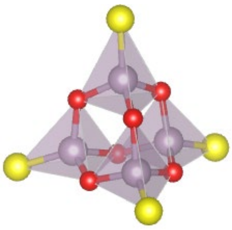	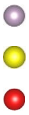	27058
3	$Ge_4S_6Br_4$	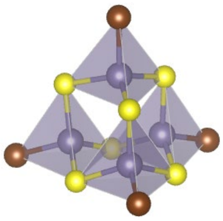	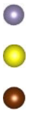	24370
3	$Si_4S_6H_4$	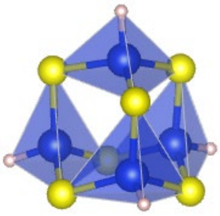	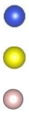	36379
4	Se_4O_{12}	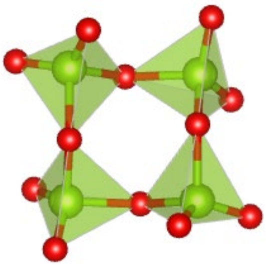	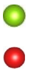	18180

4	$P_4F_8N_4$	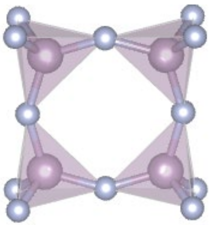	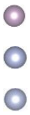	31038
4	$P_4Cl_8N_4$	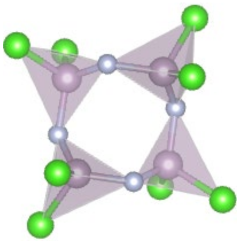	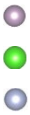	33711
4	$Si_4Cl_8O_4$	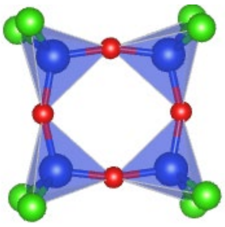	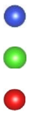	71445
5	P_4O_9	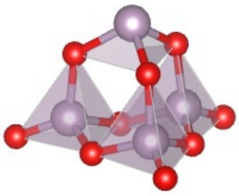	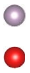	27434
5	P_4S_9	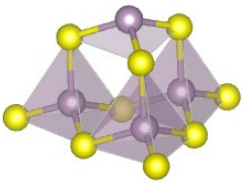	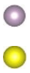	26169
5	$P_4S_6O_3$	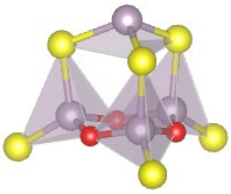	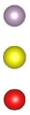	86494
5	$P_4Se_3O_6$	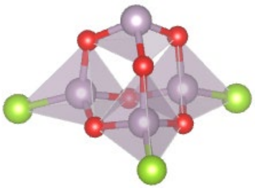	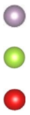	404499
6	SiI_4	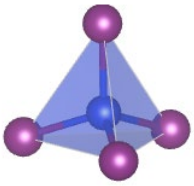	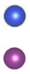	22100

6	PF_3O	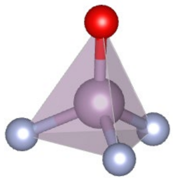	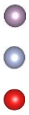	250498
6	$CrCl_2O_2$	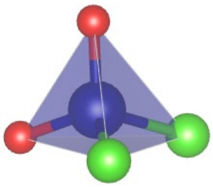	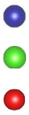	416750
7	$P_3Br_6N_3$	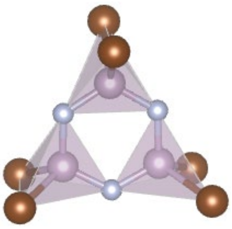	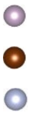	109142
7	$P_3Cl_6N_3$	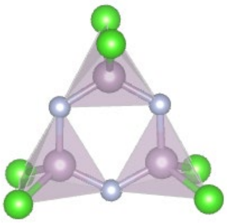	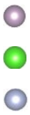	109272
7	$P_3F_6N_3$	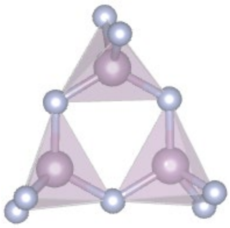	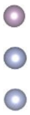	26649
8	B_2F_4	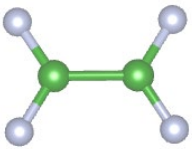	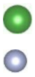	27867
8	B_2Cl_4	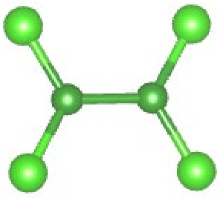	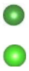	14213
8	P_2I_4	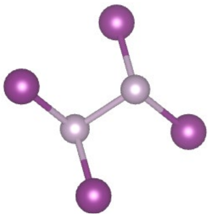	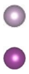	203216

9	P_4S_3	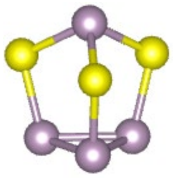	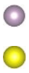	406048
9	As_4S_3	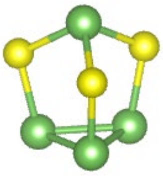	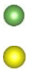	16105
9	As_4Se_3	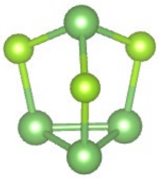	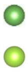	611376
10	P_4O_7	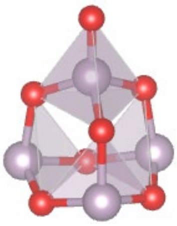	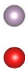	16321
10	P_4SO_6	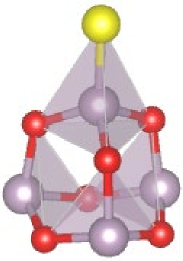	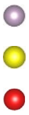	69977
10	P_4SeO_6	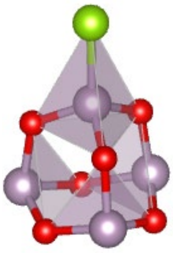	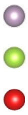	78782
11	$S_3F_3N_3$	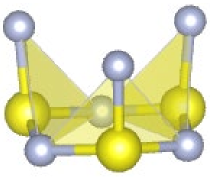	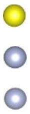	21015

11	$S_2CCl_3N_3$	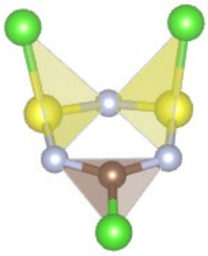	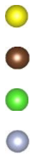	86482
11	$SC_2Cl_3N_3$	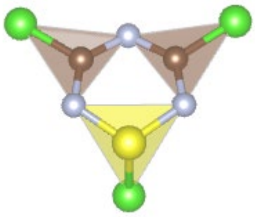	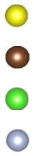	86483
12	$Sn_2P_4Cl_{16}O_6$	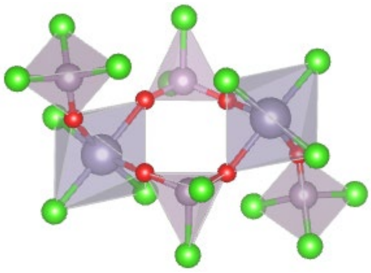	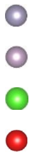	26182
12	$V_2P_4Cl_{14}O_8$	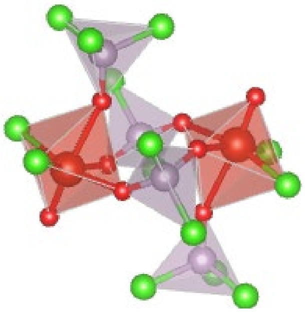	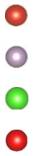	420125
12	$Ti_2P_4Cl_{16}O_6$	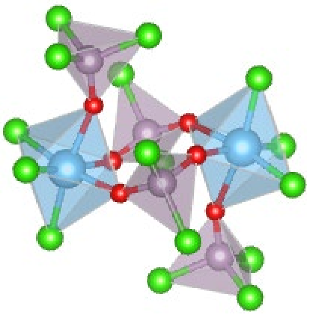	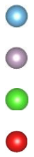	65139

Table S5. The formula, structure, and ICSD number (parent bulk crystal) of 0D boron compounds.

Formula	Structure	Element	ICSD
B_2F_4	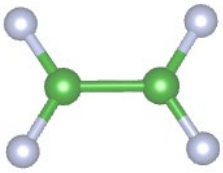	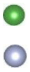	27867
B_2Cl_4	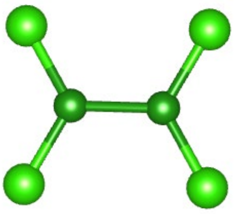	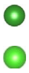	14213
$B_2O_4H_4$	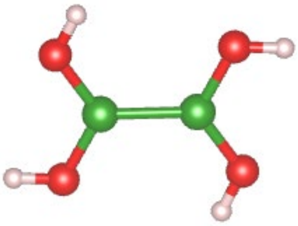	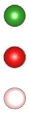	170953
$B_3N_3H_6$	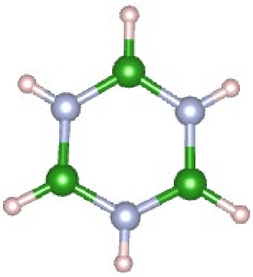	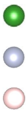	401085
B_4Cl_4	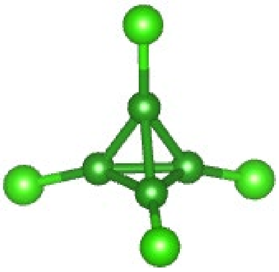	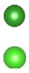	27872
B_4PF_9	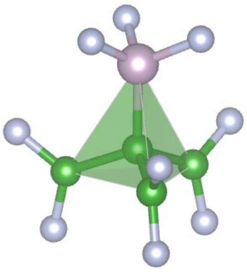	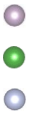	15215

B_4H_{10}	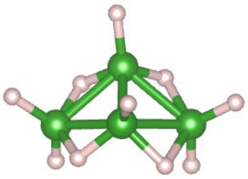		31695
B_5H_9	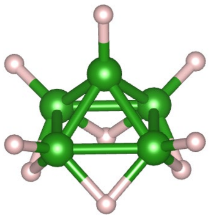		24636
B_6H_{10}	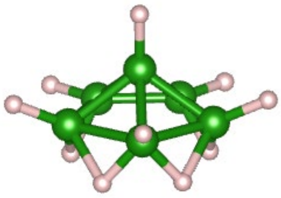		43253
B_8S_{16}	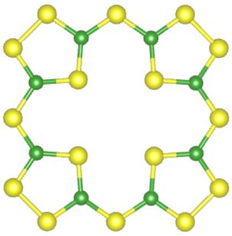		15267
$B_{10}H_{14}$	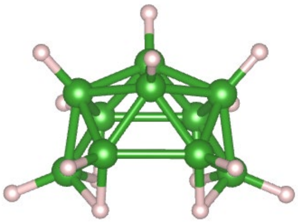		31566
$B_{10}F_{12}$	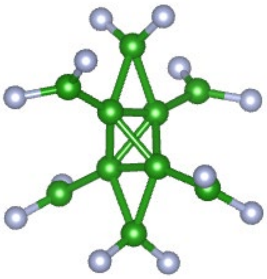		412618
$B_{10}Br_2H_{12}$	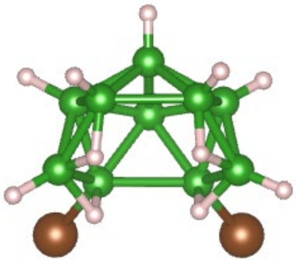		2303

$B_{12}Cl_{12}$	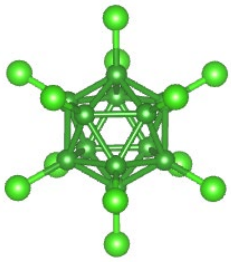	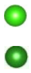	421891
$B_{12}O_{12}H_{14}$	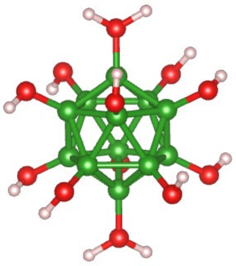	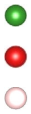	170318
$B_{18}H_{22}$	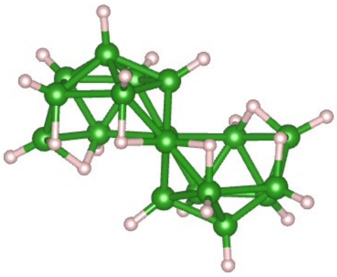	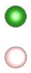	15802
$B_{18}H_{22}$	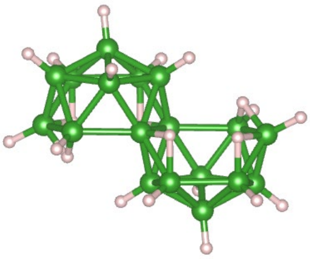	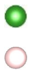	15823
$B_{18}S_2H_{16}$	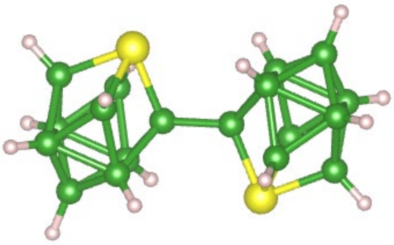	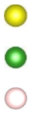	4003
$B_{20}H_{26}$	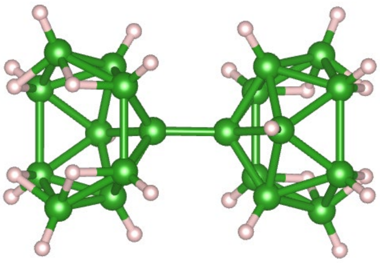	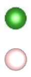	279587

Table S6. The formula, structure, and ICSD number (parent bulk crystal) of fullerene-like carbon compounds.

Formula	Structure	Element	ICSD
C_{60}	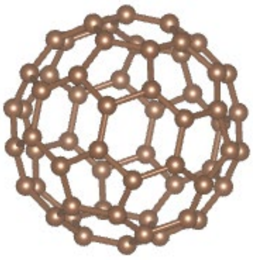	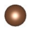	602518
$C_{60}F_{18}$	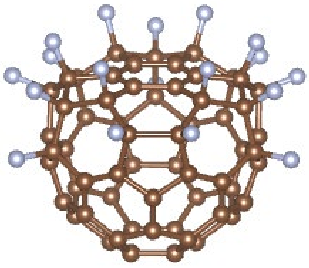	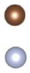	95694
$C_{64}Cl_4$	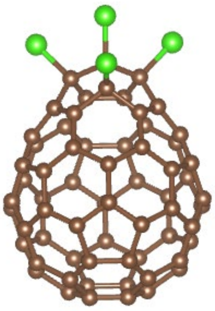	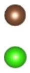	249692
$C_{68}OF_{20}$	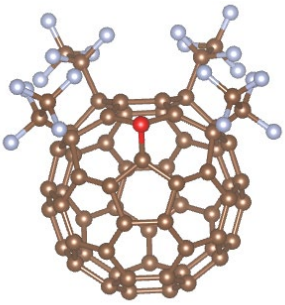	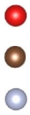	249217
$C_{68}F_{24}$	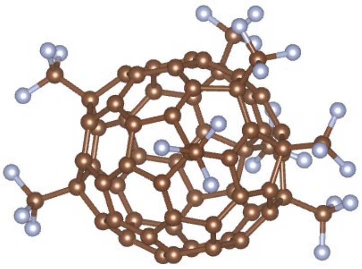	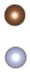	249154

$C_{70}F_{30}$	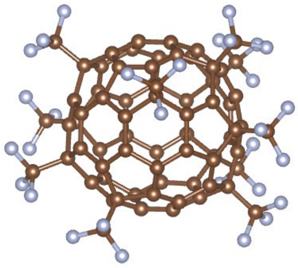	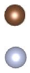	249331
$C_{76}Cl_{28}$	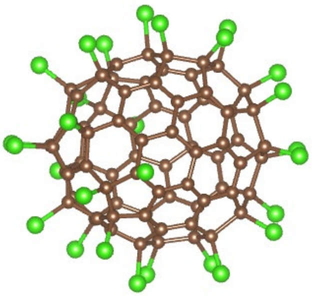	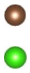	182903
$C_{78}Cl_{18}$	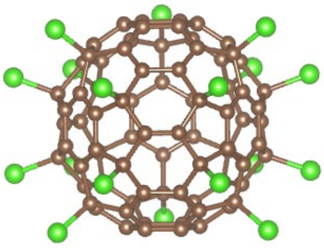	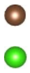	249729
$C_{80}Cl_{12}$	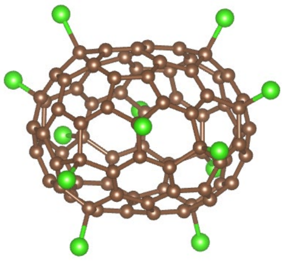	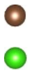	249788
$C_{90}F_{60}$	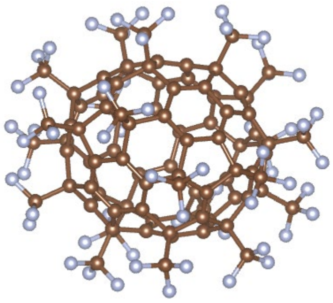	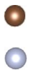	263786
$C_{108}Cl_{12}$	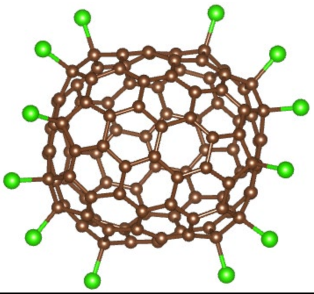	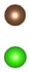	252548

Table S7. The formula, structure, and ICSD number (parent bulk crystal) of 0D phosphorus compounds.

Formula	Structure	Element	ICSD
P_4O_{10}	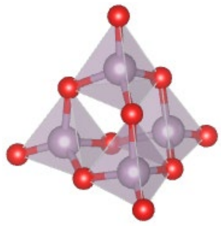	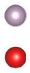	16610
P_4S_{10}	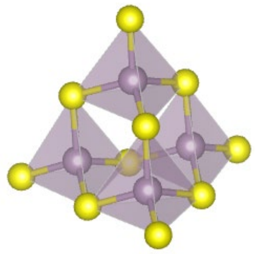	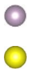	174009
$P_4S_4O_6$	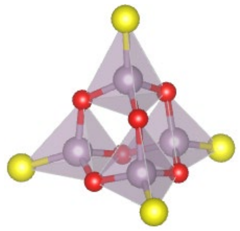	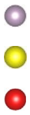	27058
P_4S_9	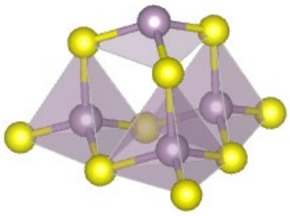	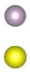	26169
P_4O_9	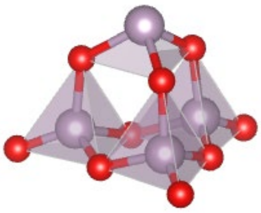	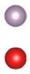	27434
$P_4S_6O_3$	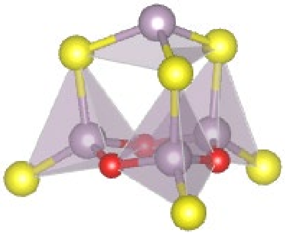	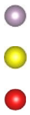	86494

P_4S_3	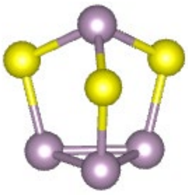	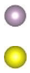	406048
P_4O_7	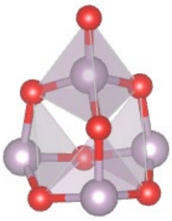	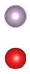	16321
P_4SO_6	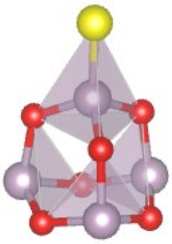	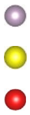	69977
P_4S_5	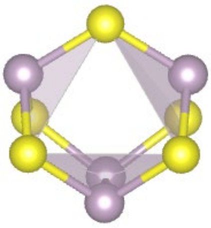	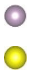	1995
P_4O_6	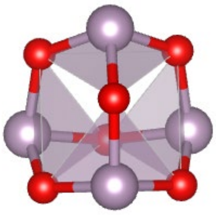	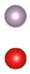	24407
P_4S_7	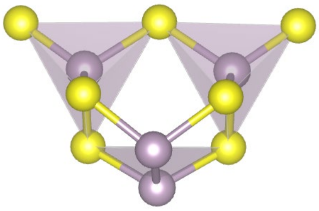	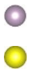	23842
P_4O_8	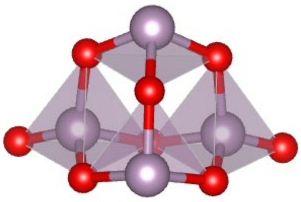	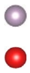	406625

Table S8. Electronic band gap of 1D compounds.

Formula	ICSD number of the parent bulk crystal	Electronic band gap
BrBi	1560	No band gap
$H_8C_4N_6S_4Fe$	10452	No band gap
O_4SCo	18176	No band gap
Cl_3Ru	22090	No band gap
I_3Hf	23947	No band gap
As_5Te_7I	31877	No band gap
$H_5N_2Cl_3Cu$	49736	No band gap
$RhBi_3$	58853	No band gap
$NbTe_4$	61113	No band gap
Se_3Nb	71498	No band gap
NbI_3	109145	No band gap
$C_4O_4F_6Ru$	171337	No band gap
$NiBi_3$	180771	No band gap
$CoBi_3$	193272	No band gap
$NiBi_3$	391336	No band gap
SCl_3Re	429458	No band gap
Se_4Nb	645375	No band gap
$CrTe_4Ta_4$	659267	No band gap
$FeTe_4Ta_4$	659268	No band gap
$NiTe_4Ta_4$	659270	No band gap
$FeNb_4Te_4$	659272	No band gap
Cl_3Zr	23163	0.0004
$SiNb_4Te_4$	659271	0.0022
NS	4025	0.0045
SeIn	32714	0.0048
$SiTe_4Ta_4$	40207	0.0132
$Sc_{12}Br_{16}Ir_3$	421533	0.0147
NCl_8Mo_2	412397	0.0157
Br_3Zr	165302	0.0202
F_4Cr	78778	0.0448

OCl_8Mo_2	65309	0.0685
$H_{21}C_9N_{15}O_3Co_2$	253962	0.0838
Br_4Os	61042	0.0840
Cl_3Ti	26069	0.1192
TiI_3	173784	0.1720
F_5Cr	419661	0.1749
F_4Cr	412032	0.1840
ZrI_3	23946	0.2136
NCl_8W_2	415768	0.2273
$N_2Cl_{11}Mo_3$	412398	0.2346
Br_3Ru	28119	0.2483
$GeZrTe_4$	240906	0.2576
$H_{20}C_4N_4MnTe_2$	426735	0.3923
Br_3Tc	260162	0.4079
Br_4W	423461	0.4092
$F_{10}CrSb$	419662	0.4157
Al_2Cl_8Ti	39565	0.4214
Cl_4W	165263	0.4285
S_3TiGe	263128	0.4311
$H_8N_4Cl_2Mn$	25784	0.4654
As_2Te_3	18208	0.4744
O_2F_3Os	240330	0.4999
OI_3W	65183	0.5166
$H_6O_6P_3Fe$	171138	0.5200
$OTi_4Te_9I_4$	82142	0.5248
OCl_3W	416393	0.5460
NbI_4	23916	0.5567
As_2Se_3	611373	0.5653
Te_4ITa	67533	0.5977
Al_2Cl_8Co	22143	0.6134
NbI_5	25503	0.6411
$TiGeTe_6$	73014	0.6682
$H_6N_2Cl_2Cu$	180189	0.6858

$NbTeI_3$	35377	0.6984
Cl_4Os	1165	0.7141
Cl_4Re	10293	0.7372
$O_4F_{12}S_2CoSb_2$	411789	0.7624
$N_3S_2VBr_2$	61088	0.7944
OF_4Os	417245	0.8143
$SeNbI_3$	410743	0.8305
Br_3Mo	413690	0.8466
Br_3NbTe	35376	0.8622
I_4Pt	15173	0.8672
$H_5BO_6Cu_2$	54883	0.8768
$H_6CN_2O_3Cu$	2779	0.9005
Al_2Cl_8Cr	416836	0.9010
$N_3S_2Cl_2V$	30742	0.9049
$H_{16}N_9Al_2Ca$	23464	0.9304
$H_{10}N_4O_8S_2Cr$	279585	0.9454
$SeBr_3Nb$	35375	0.9536
MoI_3	242166	0.9704
$H_8C_6N_6O_2Cu$	156682	0.9716
Br_4Tc	260163	0.9843
$H12N_2O_6CuSn$	103	0.9893
Cl_3SeNb	172519	1.0171
$H_{10}N_4O_8S_2Fe$	240720	1.0418
Br_4Nb	239640	1.0446
N_2O_2CoI	15286	1.0904
Cl_4Tc	26055	1.1161
I_3Re	25114	1.1306
S_4V	16797	1.1317
Sb2Se3	16680	1.1446
S_3InSb	300207	1.1496
Cl_2NbI_2	407489	1.1728
Cl_4Nb	1010	1.1808
Te_2I	107	1.2015

SbTeI	31355	1.2084
OBr_4W	49547	1.2119
Se_9Nb_2	62538	1.2119
$Cl_7Se_5Nb_3$	10066	1.2439
P_4CuSe_3I	422534	1.2545
C	412242	1.3039
$Cl_3Se_{10}Nb_3$	240715	1.3129
$Se_{10}Br_3Nb_3$	201731	1.3322
Cl_4Ta	402406	1.3335
S_3ZrSn	73711	1.3366
Sb2S3	171850	1.3859
Cl_2I_2Ta	69688	1.3872
$S_3ClCuAs_4$	419754	1.4387
CCl_8W_2	412384	1.4921
NCl_3VI	10470	1.4962
Cl_2Te_3	105	1.5191
Te_6I_3Ir	423670	1.5200
TeI	108	1.5276
S_3GePd	408505	1.5278
PSnI	430054	1.5319
S_2Cl_3Mo	28062	1.5321
NCl_4V	28128	1.5412
P_4S_3CuI	418076	1.5461
$Br_{10}PtBi_6$	418335	1.5490
P_4S_3CuBr	421076	1.5528
$RhTe_6I_3$	423671	1.5793
SeSbI	31292	1.6270
S_3SnHf	65667	1.6292
$H_4C_3O_2ClCu$	20522	1.6587
S_3Sn_2	15338	1.6681
$H_{12}C_4O_{15}S_4V_2$	262179	1.7141
Br_3Te_6Ir	423673	1.7206
$PS_7ClCuAs_7$	262251	1.7357

Br_3RhTe_6	423669	1.7434
$Cl_{10}PtBi_6$	418334	1.7441
Al_2Cl_8V	415951	1.7605
OBr_3Nb	418089	1.7936
Cl_3RhTe_6	422367	1.8057
NCl_3Mo	15117	1.8262
Cl_3Te_6Ir	423672	1.8555
AsSeI	200799	1.8829
PS_7CuAs_7Br	262252	1.9142
SeBrBi	76649	1.9157
C_4O_4CoCu	47183	1.9248
SBrSb	25571	1.9424
$H_6N_2ZnSe_4$	425783	1.9491
$H_{10}C_2N_2ZnSe_4$	248322	1.9642
$H_8C_2N_4S_2BrAg$	167543	1.9757
SSbI	25572	1.9832
PS_4Sb	172038	2.0013
SSn_2I_2	207	2.0518
P	391323	2.0621
ZrI_4	8068	2.0697
O_3Cr	109366	2.0716
OCl_4W	25519	2.1000
$H_{25}C_8O_{10}NaMo_2$	192735	2.1006
$SiCl_2$	85526	2.1028
$H_{24}C_9N_6S_3AgI$	167544	2.1323
SIBi	25575	2.1396
SeInI	100704	2.1730
F_5Bi	25023	2.2037
P_2S_7	423061	2.2624
BPS_4	24618	2.3406
$H_6C_4O_2S_4Cd$	110011	2.3785
I_4Hf	200826	2.4149
$H_{10}N_4O_8S_2Mn$	249335	2.4423

PSe	74878	2.4514
$SiSe_2$	24592	2.4890
OCl_3Nb	26471	2.4984
$O_2F_{10}Te_2Hg$	194226	2.5079
N_3Cl_3Ti	15996	2.5441
$H_{12}C_4O_8P_2Mn$	249436	2.5667
N_4Si_3	67241	2.5823
$H_{12}C_3N_6S_3Br_2Ag_2$	167545	2.5959
N_2O_8Mo	421223	2.6568
$H_{10}C_6NS_2IPb$	251754	2.6701
$H_8C_4N_6S_4Cd$	170723	2.7309
HO_8S_2I	407853	2.7474
Br_2Sn	411177	2.7482
$O_{11}S_2I_2$	405696	2.7736
$H_9C_3O_4V$	163034	2.8134
ClSnI	23262	2.8164
H_8N_4ZnTe	170880	2.8246
$AlPS_4$	15910	2.8988
OF_9MoSb	201410	2.9574
$H_8N_{10}Zn$	421952	2.9926
$H_8C_2N_4O_8S_2CdRe_2$	170223	3.0121
$H_9C_7O_4F_4S_2Ag$	249365	3.0390
O_2Se	24022	3.0659
$H_{10}C_2O_8S_2Hg$	251311	3.0667
O_7TiI_2	424632	3.0827
$H_6O_{16}S_4Mn$	408754	3.0907
$O_3F_2Te_2$	82162	3.0919
O_5Se_2	10471	3.1568
$H_{16}C_7O_3F_3SiS_3Ag$	249786	3.2233
H_3CCl_3Te	281531	3.3109
SiS_2	26858	3.3537
Cl_2Sn	15452	3.3572
C_2O_4Sn	54909	3.3603

$H_{19}C_7Si_2Br_2In$	163067	3.4166
$H_2F_{14}As_2Hg$	429284	3.4281
$H_{27}LiC_9O_3Si_4$	173212	3.4361
O_3FRe	415418	3.5276
H_9C_3IPb	405865	3.5503
$H_4CN_2O_4Mo$	249697	3.6055
O_2F_8SbRe	280248	3.6349
$H_{12}C_4O_2NaS_2Br$	249333	3.6676
$O_{14}S_4Zr$	424062	3.7054
$F_{13}KSb_4$	24740	3.7086
$O_6MgP_4Cl_{10}$	15015	3.7793
HO_5AsTe	425500	3.7902
$F_{18}InSb_3$	421923	3.7996
Cl_4Zr	26049	3.8491
F_4Sb	200035	3.8518
$H_8N_4Cl_2Zn$	15875	3.9056
FClSn	647	3.9545
$H_{10}N_4O_8S_2Cd$	240376	4.0207
$C_6O_6F_9Sc$	196174	4.0309
Cl_6GaSb	24786	4.1015
$H_6O_{16}S_4Cd$	40438	4.1848
$F_{10}NbSb$	16095	4.1971
$H_4C_2N_4Cl_2Zn$	240736	4.2005
Cl_4Hf	402054	4.3463
OFSb	19019	4.3786
$H_{14}C_4NSi_2K$	180206	4.4316
$H_{10}C_3OSn$	252900	4.4408
$H_{12}O_{12}P_6CaSn$	188683	4.4426
C_3N_3As	35330	4.4601
$H_7C_2O_3ClSeSn$	782622	4.5156
F_4Ti	78737	4.5169
BeI_2	92585	4.5414
$H_6C_2N_4O_4Zn$	9202	4.5500

OF_2Te	88415	4.5864
F_4Te	85452	4.6018
$F_{22}Ta_4Hg$	431295	4.6358
$H_{18}B_3N_2Y$	251866	4.6483
$H_3C_3O_6Sc$	281595	4.6652
$H_{10}C_3NAl$	380172	4.7003
$H_{11}C_3OAlSi$	172440	5.0792
$H_6O_{16}S_4Zn$	404089	5.2954
O_3S	24723	5.5954
$MgAl_2Cl_8$	62046	5.6689
O_2Si	25632	5.7574
$BeBr_2$	92584	5.8106
NF_2P	9684	6.3141
$H_6O_{16}MgS_4$	39276	6.3451
$F_{22}CdTa_4$	431293	6.4633
$BeCl_2$	31696	6.8519

References

1. Pauling L. *The Nature of the Chemical Bond and the Structure of Molecules and Crystals: An Introduction to Modern Structural Chemistry*. Ithaca, NY: Cornell University Press, 1960.
2. Slater JC. Atomic radii in crystals. *J Chem Phys* 1964; **41**: 3199-3204.
3. Shannon RD. Revised effective ionic radii and systematic studies of interatomic distances in halides and chalcogenides. *Acta Crystallogr Sect A* 1976; **32**: 751-767.
4. Larsen PM, Pandey M, Strange M *et al*. Definition of a scoring parameter to identify low-dimensional materials components. *Phys Rev Mater* 2019; **3**: 034003.
5. Mounet N, Gibertini M, Schwaller P *et al*. Two-dimensional materials from high-throughput computational exfoliation of experimentally known compounds. *Nat Nanotechnol* 2018; **13**: 246-252.
6. Silvi B & Savin A. Classification of chemical bonds based on topological analysis of electron localization functions. *Nature* 1994; **371**: 683-686.
7. Becke AD & Edgecombe KE. A simple measure of electron localization in atomic and molecular systems. *J Chem Phys* 1990; **92**: 5397-5403.
8. Savin A, Jepsen O, Flad J *et al*. Electron localization in solid-state structures of the elements: the diamond structure. *Angew Chem Int Edit* 1992; **31**: 187-188.
